# Explainable XGBoost model and nomogram for risk factor identification and risk prediction in cerebral small vessel disease: a machine learning-based retrospective cohort study

**DOI:** 10.3389/fneur.2026.1786279

**Published:** 2026-07-24

**Authors:** Xi Zhu, Xuhui Liu, Xujie Wang, Yuyi Fan, Ming Chen

**Affiliations:** 1 Department of Neurology, The Fifth Affiliated Hospital of Xinjiang Medical University, Ürümqi, China; 2 Department of Neurology, The Second Hospital of Lanzhou University, Lanzhou, Gansu, China; 3 Department of Emergency ICU, The Affiliated Hospital of Qinghai University, Xining, Qinghai, China; 4 Department of Emergency, The Fifth Affiliated Hospital of Xinjiang Medical University, Ürümqi, China

**Keywords:** cerebral small vessel disease, machine learning, XGBoost, SHAP, nomogram, risk prediction

## Abstract

**Background:**

Cerebral small vessel disease (CSVD) is a common, clinically significant vascular disorder that frequently leads to cognitive impairment, dementia, and poor overall prognosis. Owing to its complex hemodynamic characteristics and multifactorial pathophysiology, early identification of individuals at high risk for CSVD remains a clinical challenge. This study aimed to develop and validate an interpretable machine learning (ML) model for predicting the occurrence of CSVD.

**Methods:**

We retrospectively enrolled 1,640 adult patients treated at the Fifth Affiliated Hospital of Xinjiang Medical University between September 2019 and December 2024. Twenty-three candidate variables (demographics, vitals, biomarkers, comorbidities) were evaluated. Feature selection was performed using least absolute shrinkage and selection operator (LASSO) regression, followed by stepwise backward elimination in multivariable logistic regression. Six supervised ML algorithms (DT, KNN, LR, LightGBM, XGBoost, SVM) were compared. Performance was assessed using ROC curves, calibration plots, and decision curve analysis (DCA). The optimal model was interpreted using SHapley Additive exPlanations (SHAP), and a bedside clinical nomogram was constructed.

**Results:**

Ten independent predictors were identified: blood glucose, history of hypertension, systolic blood pressure, age, triglycerides, history of stroke, cystatin C, C-reactive protein, homocysteine, and body mass index. Among all models, XGBoost demonstrated the best performance, with an AUC of 0.968 in the training cohort and 0.938 in the validation cohort. Calibration plots and DCA confirmed its clinical utility. The derived nomogram demonstrated strong prognostic discrimination (*p* < 0.0001). The XGBoost model achieved an accuracy of 88.0%, sensitivity of 80.9%, specificity of 93.8%, and an F1 score of 0.86, corresponding to a 5.4-percentage-point gain in AUC over logistic regression. Ten-fold cross-validation confirmed this ranking, with a mean AUC of 0.934 ± 0.016.

**Conclusions:**

We validated an interpretable XGBoost-based ML model that facilitates early risk stratification and targeted interventions for CSVD. Because the model relies only on routinely collected, low-cost variables and open-source software, it is readily transferable to resource-limited settings; future work will focus on prospective, multicentre external validation and on embedding the nomogram into electronic-health-record decision support.

## Introduction

Cerebral small vessel disease (CSVD) encompasses a spectrum of pathological processes affecting intracranial small vessels and microvasculature, including small arteries, veins, and capillaries, arising from genetic or acquired etiologies and leading to diverse clinical, cognitive, neuroimaging, and neuropathological manifestations (1, 2). CSVD demonstrates a strong age-related pattern, with prevalence increasing in parallel with population aging in China. Progressive accumulation of CSVD burden is associated with impairments in cognition, motor function, emotional regulation, and bladder or bowel control (3).

With the global acceleration of demographic aging, the incidence of CSVD has increased substantially, resulting in a growing public health burden. Current estimates suggest that CSVD accounts for roughly one-fifth of all cerebrovascular diseases and represents a predominant pathological substrate underlying vascular dementia (approximately 40%) and lacunar infarction (approximately 25%) (4). Secondary prevention remains the cornerstone for reducing stroke recurrence, disability, and mortality. Beyond non-modifiable factors such as age and sex, multiple modifiable risk factors—including hypertension, diabetes, dyslipidemia, cardiac comorbidities, smoking, and alcohol intake—contribute significantly to disease progression. Accordingly, timely identification of patients at elevated risk of stroke recurrence is essential for effective secondary prevention. Despite advances in neuroimaging that have improved CSVD severity assessment, precise risk stratification for adverse outcomes in distinct CSVD subpopulations continues to present a clinical challenge.

Conventional statistical approaches applied to high-dimensional clinical data in CSVD are often constrained by methodological complexity and limited predictive performance. In contrast, machine learning (ML) techniques have gained increasing attention in stroke research due to their ability to capture nonlinear relationships and complex feature interactions without reliance on prespecified assumptions (5, 6). Among these, tree-based ensemble algorithms—particularly extreme gradient boosting (XGBoost)—have demonstrated strong predictive accuracy and robustness across a range of medical applications, including cardiovascular disorders, sepsis, and stroke outcome prediction (7, 8). Furthermore, recent developments in explainable artificial intelligence (XAI), such as Shapley Additive Explanations (SHAP), allow for transparent attribution of feature-level contributions to model outputs, thereby improving interpretability and facilitating clinical translation of complex models (9, 10). However, the application of interpretable ML frameworks for risk prediction in CSVD populations remains limited. The most recent efforts in this field have largely been confined either to small single-modality imaging cohorts or to narrow phenotypes. For example, interpretable ML pipelines have been built for CSVD risk screening in community samples (11), for CSVD-related depression using multimodal MRI (12), and for white-matter-hyperintensity burden using LASSO-based nomograms (13, 14), while ensemble learners with SHAP have been proposed as auxiliary diagnostic systems for CSVD (15). Tree-based gradient boosting has likewise become the dominant performer in adjacent cerebrovascular tasks such as intracerebral-hemorrhage prognosis (16, 17) and acute-ischaemic-stroke functional prognosis (14). Nevertheless, a parsimonious, fully interpretable model that couples XGBoost, SHAP attribution and a bedside nomogram, derived entirely from routinely available clinical and laboratory variables in a large hospital-based CSVD cohort, has not yet been reported.

The present study sought to develop and validate an interpretable and reliable predictive model for CSVD using routinely collected clinical and laboratory parameters. A total of 1,640 patients from a single tertiary care center were retrospectively included, and 23 candidate predictors were initially evaluated. Feature selection was performed through a multi-step procedure incorporating least absolute shrinkage and selection operator (LASSO) regression followed by stepwise logistic regression. Six supervised ML models were subsequently constructed and compared. Based on comprehensive assessment of discrimination, calibration, and clinical utility, XGBoost was selected as the optimal model. SHAP analysis was employed to identify key predictive features, which were then integrated into an intuitive nomogram to support individualized risk stratification and early intervention in this high-risk stroke population.

## Methods

### Study design and population

This retrospective observational study was conducted at the Fifth Affiliated Hospital of Xinjiang Medical University. The study protocol was reported in accordance with the Guidelines for Reporting Observational Studies Using Routinely Collected Health Data (RECORD). Ethical approval was obtained from the Institutional Review Board of the Fifth Affiliated Hospital of Xinjiang Medical University (Approval No. XYDWFYLSk-2025-229). Owing to the retrospective and de-identified nature of the data, the requirement for informed consent was waived. All procedures complied with national regulations and institutional standards regarding patient privacy and data confidentiality.

### Inclusion and exclusion criteria

**Figure 1:**
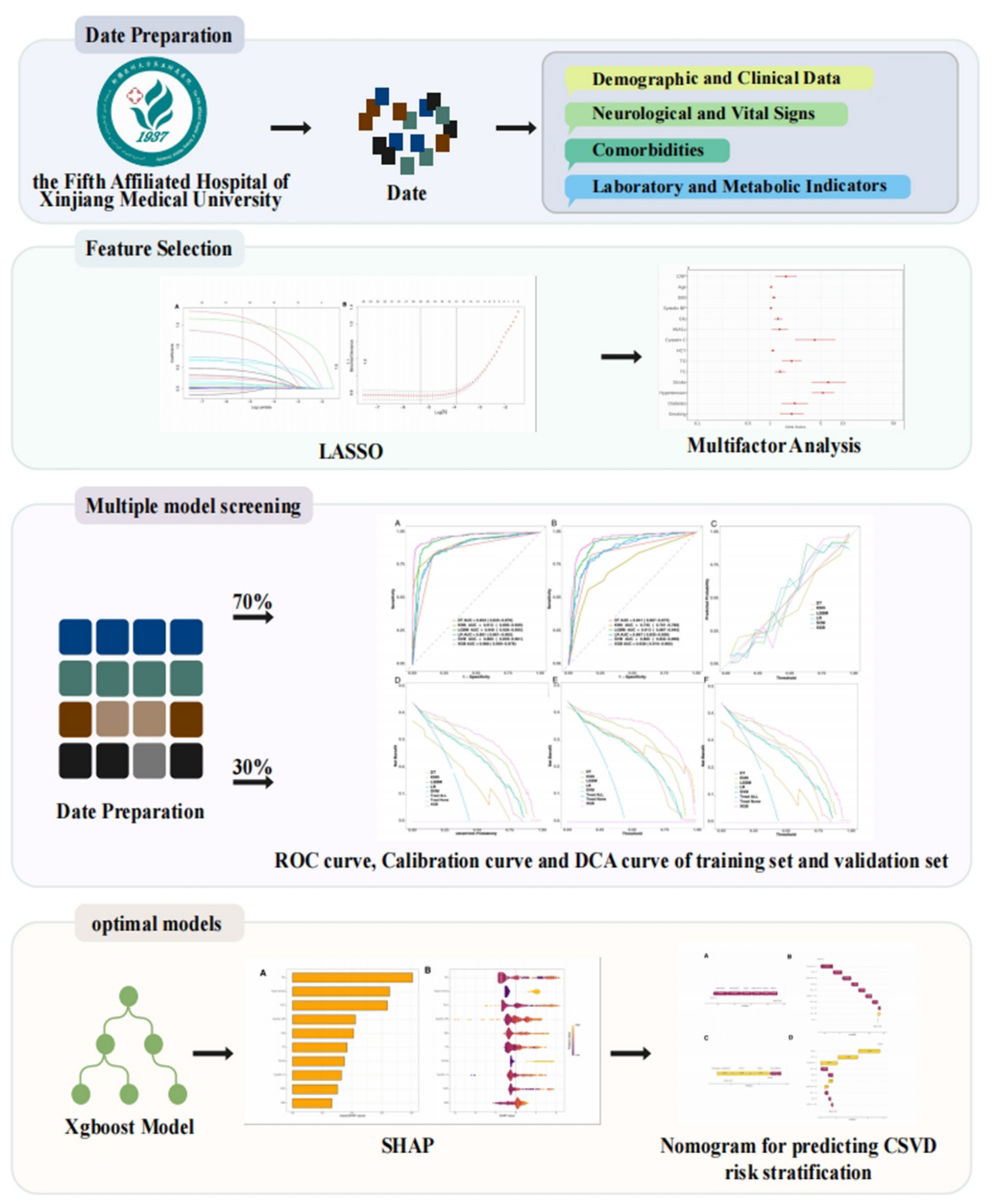
Flowchart of this study.

We consecutively enrolled adult inpatients (aged ≥18 years) hospitalized between September 2019 and December 2024 who met the following inclusion criteria:(1) age ≥18 years;(2) availability of brain magnetic resonance imaging (MRI) and fulfillment of the diagnostic criteria for cerebral small vessel disease (CSVD) according to the Chinese Consensus on the Diagnosis and Treatment of Cerebral Small Vessel Disease (2020);(3) presence of at least one CSVD-related imaging feature on MRI, including white matter hyperintensities, lacunar infarctions, enlarged perivascular spaces, or cerebral microbleeds.The exclusion criteria were as follows:(1) concomitant shock, severe infectious diseases, or severe cardiac, hepatic, renal, or pulmonary diseases;(2) sepsis or autoimmune diseases;(3) pregnancy or lactation;(4) use of medications within 1 month prior to admission that could significantly affect laboratory test results. A total of 1,640 eligible patients were identified and randomly divided into a training cohort (*n* = 1,148) and a validation cohort (*n* = 492) at a 7:3 ratio using stratified sampling. The patient selection process is illustrated in Figure 1. A complete numerical description of every candidate feature—including data type, measurement unit, full descriptive statistics [median (Q1, Q3) or *n* (%)] and the univariable comparison between patients with and without CSVD—together with the overall sample size and the class-balanced training/validation partition is provided in Supplementary Tables S1, S2.

### Candidate predictors

Based on a comprehensive literature review and clinical relevance, 23 candidate predictors were selected: (1) Demographic variables: sex, age, body mass index (BMI), smoking history, and alcohol consumption; (2) Vital signs: systolic blood pressure (SBP) and diastolic blood pressure (DBP); (3) Comorbidities: hypertension, diabetes mellitus, and stroke; (4) Laboratory parameters: blood glucose (Glu), glycated hemoglobin (HbA1c), D-dimer, fibrinogen (FIB), blood urea nitrogen (BUN), uric acid, creatinine, cystatin C, homocysteine (HCY), C-reactive protein (CRP), low-density lipoprotein cholesterol (LDL), triglycerides (TG), and total cholesterol (TC).

### Statistical analysis and feature selection

Continuous variables are presented as medians with interquartile ranges (IQRs), while categorical variables are expressed as counts and percentages. Between-group comparisons were performed using the Mann–Whitney U test for continuous variables and the chi-square test or Fisher's exact test for categorical variables. Candidate predictors with p < 0.05 in univariate analyses were subjected to dimensionality reduction using least absolute shrinkage and selection operator (LASSO) regression with 10-fold cross-validation. The optimal penalty parameter was selected according to the one-standard-error rule (λ = 0.20), yielding 14 variables with non-zero coefficients. These variables were subsequently entered into a multivariable logistic regression model with backward stepwise selection based on the Akaike information criterion (AIC). Multicollinearity was assessed using the variance inflation factor (VIF), with all predictors showing VIF values < 3.2, indicating no significant collinearity. Fourteen independent predictors were retained, after which recursive feature elimination combined with 5-fold cross-validation was applied to identify the top 10 predictors: blood glucose (Glu), homocysteine (HCY), hypertension, triglycerides (TG), stroke, systolic blood pressure (Systolic_BP), age, cystatin C (Cystatin_C), C-reactive protein (CRP), and body mass index (BMI).

### Model construction and evaluation

Based on 10 selected predictors, six supervised machine learning models were developed, including decision tree (DT), k-nearest neighbors (KNN), logistic regression (LR), Light Gradient Boosting Machine with histogram-based algorithm (LGBM), extreme gradient boosting (XGBoost), and support vector machine (SVM). Model training was conducted using 10-fold cross-validation within the training cohort, while performance evaluation was performed in an independent validation cohort. Model performance was assessed across three principal dimensions. Discriminative ability was evaluated using the area under the receiver operating characteristic curve (AUC), ROC curves, precision–recall curves, sensitivity, specificity, accuracy, F1 score, positive predictive value (PPV), and negative predictive value (NPV). Calibration performance was examined using calibration plots, the Hosmer–Lemeshow goodness-of-fit test, and Brier scores. Clinical utility was assessed via decision curve analysis (DCA) across a range of threshold probabilities. Among all evaluated models, XGBoost consistently demonstrated superior performance across discrimination, calibration, and clinical utility metrics and was therefore selected as the final predictive model. The complete analytical pipeline—from cohort assembly and feature selection to model comparison, interpretation and nomogram construction—is summarized as a flowchart in Supplementary Figure S1, and the corresponding algorithmic steps are additionally organized as a schematic mind-map in Supplementary Figure S2 (15). To minimize the risk of overfitting, hyperparameters for every algorithm were tuned and all models were evaluated using stratified ten-fold cross-validation; the full hyperparameter grid and final settings for all six models are reported in Supplementary Table S3, and the additive-tree architecture of the selected XGBoost model is depicted in Supplementary Figure S3.

### Model interpretability and nomogram construction

To enhance clinical interpretability, the XGBoost model was further analyzed using Shapley Additive Explanations (SHAP). For individualized, sample-level interpretation, SHAP beeswarm plots were generated separately for positive and negative samples. To rigorously evaluate the performance of the nomogram, a dual-layer validation strategy was implemented. First, probability-based model rationality was assessed within the XGBoost framework by constructing variable-specific ROC curves and DCA curves, along with additional evaluations based on predicted probability metrics. Second, probability analyses based on the nomogram-derived score (nomoScore) were conducted by generating bar charts and box plots across different risk strata in both the training and validation cohorts. Finally, a forest plot was constructed according to established diagnostic evaluation criteria for the nomoScore. A nomogram was subsequently developed using the ten identified predictors, in which weighted scores were assigned to each variable to generate an aggregate score (nomoScore). This composite score corresponded to the predicted probability of adverse outcomes. Logistic regression analysis confirmed that the nomoScore served as an independent prognostic factor, and its diagnostic performance was quantified by calculating sensitivity, specificity, PPV, NPV, and the Youden index. All statistical analyses were performed using R software (version 4.2.1) and Python (version 3.6.5), with a two-sided *p* value < 0.05 considered statistically significant. All computations were executed on a standard commodity workstation using exclusively open-source, freely licensed software (Python, R and their scientific libraries); the detailed hardware/software configuration and the associated acquisition cost are itemized in Supplementary Table S4. For each algorithm we additionally profiled wall-clock training time, per-cohort inference latency, peak training memory and serialized model size so that computational efficiency could be compared alongside predictive performance.

## Results

A total of 1,640 patients were included in the study, with a median age of 65 years (interquartile range, 61–71 years). The cohort comprised 872 men (53.17%) and 768 women (46.83%). Among these, 1,148 patients were allocated to the training cohort and 492 to the validation cohort. As summarized in Table 1, baseline characteristics were generally comparable between the two cohorts. The median age did not differ significantly between the training and validation cohorts [65.00 (61.00–71.00) vs. 66.00 (61.00–71.00), *p* > 0.05)] No statistically significant differences were observed in other key variables, including glycemic and lipid parameters, renal function markers, inflammatory indices, or vascular risk factors such as hypertension, diabetes mellitus, smoking, and alcohol consumption (all *p* > 0.05), indicating satisfactory baseline balance for model development and validation. Comparative analyses between the CSVD and non-CSVD groups revealed several significant differences (Supplementary Table S5). Patients in the CSVD group exhibited a higher prevalence of smoking (32.10% vs. 21.04%, *p* < 0.001) and were more likely to have a history of stroke (24.32% vs. 75.68%, *p* < 0.001), hypertension (54.37% vs. 12.89%, *p* < 0.001), and diabetes mellitus (28.14% vs. 9.91%, *p* < 0.001). In addition, CSVD patients demonstrated significantly elevated levels of homocysteine, lipid profiles, and coagulation-related parameters.

**Table 1:** Baseline characteristics of the study population in the training and validation cohorts.

Characteristics	[ALL] *N* = 1,640	Test *N* = 492	Train *N* = 1,148	*p*. overall
Grouped	0.446 (0.497)	0.443 (0.497)	0.448 (0.497)	0.862
CRP	0.880 [0.550; 1.210]	0.865 [0.560; 1.190]	0.880 [0.548; 1.220]	0.921
BUN	5.030 [4.180; 5.610]	4.990 [4.168; 5.525]	5.060 [4.190; 5.650]	0.372
Drink				0.779
No	1,345 (82.012%)	401 (81.504%)	944 (82.230%)	
Yes	295 (17.988%)	91 (18.496%)	204 (17.770%)	
Age	65.000 [61.000; 71.000]	66.000 [61.000; 71.000]	65.000 [61.000; 71.000]	0.691
BMI	24.002 (2.872)	23.912 (2.807)	24.040 (2.900)	0.401
Systolic_BP	131.000 [122.000; 141.000]	131.000 [122.000; 140.000]	131.000 [122.000; 141.000]	0.716
Diastolic_BP	78.760 (10.638)	78.537 (10.594)	78.855 (10.660)	0.577
Glu	5.040 [4.530; 5.680]	4.990 [4.525; 5.620]	5.045 [4.540; 5.730]	0.394
HbA1c	5.800 [5.400; 6.200]	5.800 [5.500; 6.200]	5.800 [5.400; 6.200]	0.595
D_dimer	104.000 [55.000; 149.250]	104.000 [54.000; 153.000]	104.000 [56.000; 148.000]	0.790
FIB	2.750 [2.480; 3.090]	2.745 [2.500; 3.100]	2.750 [2.470; 3.080]	0.569
Uric_acid	298.774 (77.764)	295.419 (77.906)	300.212 (77.693)	0.253
Creatinine	77.000 [66.000; 86.250]	77.000 [67.000; 87.000]	76.000 [66.000; 86.000]	0.926
Cystatin_C	0.890 [0.720; 1.070]	0.910 [0.728; 1.090]	0.880 [0.720; 1.053]	0.154
HCY	10.000 [8.000; 12.000]	9.000 [8.000; 12.000]	10.000 [8.000; 12.000]	0.798
LDL	2.456 (0.728)	2.488 (0.706)	2.442 (0.737)	0.232
TG	1.030 [0.720; 1.410]	1.020 [0.748; 1.322]	1.030 [0.700; 1.430]	0.503
TC	4.000 [3.360; 4.720]	4.010 [3.357; 4.752]	3.995 [3.367; 4.710]	0.770
Gender				0.285
Male	872 (53.171%)	272 (55.285%)	600 (52.265%)	
Female	768 (46.829%)	220 (44.715%)	548 (47.735%)	
Stroke				1.000
No	1,417 (86.402%)	425 (86.382%)	992 (86.411%)	
Yes	223 (13.598%)	67 (13.618%)	156 (13.589%)	
Hypertension				1.000
No	1,125 (68.598%)	337 (68.496%)	788 (68.641%)	
Yes	515 (31.402%)	155 (31.504%)	360 (31.359%)	
Diabetes				0.855
No	1,344 (81.951%)	405 (82.317%)	939 (81.794%)	
Yes	296 (18.049%)	87 (17.683%)	209 (18.206%)	
Smoke				0.685
No	1,214 (74.024%)	368 (74.797%)	846 (73.693%)	
Yes	426 (25.976%)	124 (25.203%)	302 (26.307%)	

CRP, C-reactive protein; BUN, blood urea nitrogen; BMI, body mass index; HbA1c, gycated hemoglobin; FIB, fibrinogen; UA, uric acid; HCY, homocysteine; LDL, low-density lipo protein; TG, triglycerides; TC, total cholesterol; Categorical variables are represented as follows: Gender: Male, Female; Hypertension: No, Yes; Diabetes: No, Yes; Smoke: No, Yes; Drink: No, Yes; Stroke: No, Yes. Statistical comparisons between groups were performed using the Mann-Whitney U test for continuous variables and the 2 test for categorical variables. A *p*-value < 0.05 was considered statistically significant.

C-reactive protein (CRP), Blood Urea Nitrogen (BUN), Body Mass Index (BMI), Glycated Hemoglobin (HbA1c), Fibrinogen (FIB), Uric Acid (UA), Homocysteine (HCY), Low-Density Lipoprotein (LDL), Triglycerides (TG), Total Cholesterol (TC), Categorical variables are represented as follows: Gender: Male, Female; Hypertension: No, Yes; Diabetes: No, Yes; Smoke: No, Yes; Drink: No, Yes; Stroke: No, Yes. Statistical comparisons between groups were performed using the Mann-Whitney U test for continuous variables and the χ^2^ test for categorical variables. A *p*-value < 0.05 was considered statistically significant.

### Feature selection using LASSO regression

**Figure 2:**
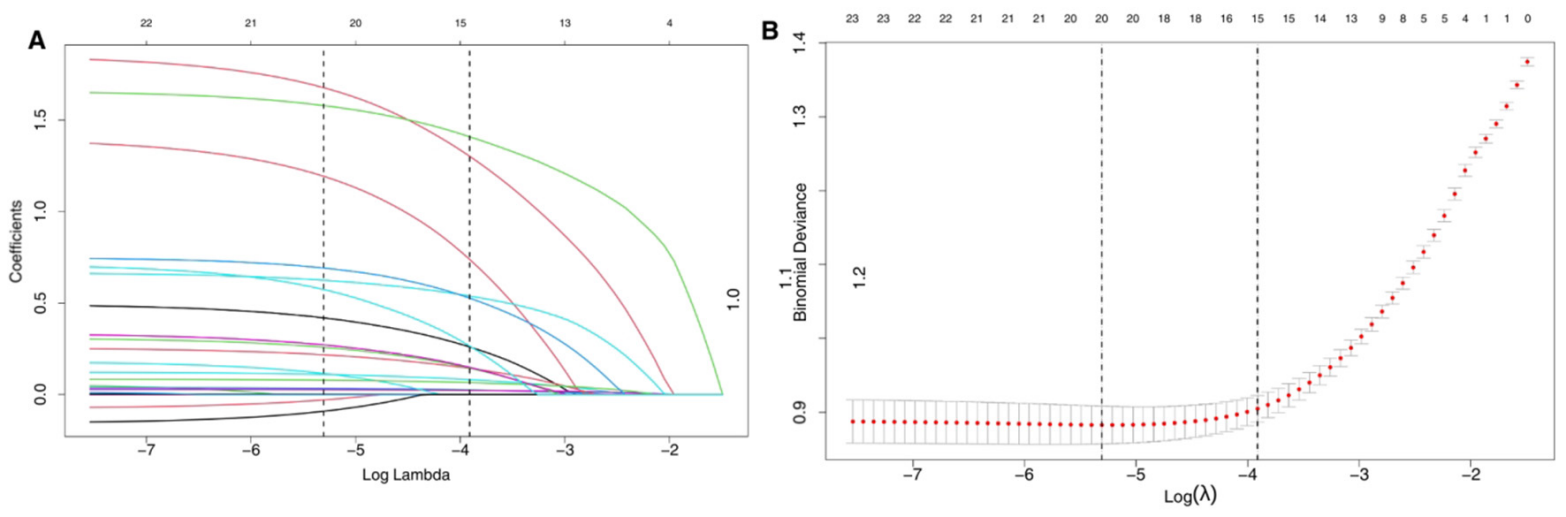
LASSO regression for feature selection. **(A)** 10-fold cross-validation curve showing binomial deviance vs. log (λ). The dotted vertical line indicates the optimal λ0.1se = 0.020 corresponding to the minimum deviance. **(B)** LASSO coefficient profiles showing the shrinkage paths of all candidate variables. The vertical dashed line represents λ. Min, at which 14 predictors retained non-zero coefficients.

To identify the most informative predictors and minimize overfitting, LASSO regression was applied to the training cohort. Using 10-fold cross-validation and the minimum binomial deviance criterion, the optimal regularization parameter was determined. As shown in Figure 2A, λ0.1se = 0.020 was selected as the optimal penalty value, resulting in 14 variables with non-zero coefficients: CRP, age, BMI, systolic blood pressure, glucose, HbA1c, cystatin C, HCY, TG, TC, stroke, hypertension, diabetes, and smoking. The coefficient shrinkage paths of all candidate variables across log (λ) values are displayed in Figure 2B, illustrating an optimal balance between model parsimony and predictive performance. The complete list of selected features and their coefficients is provided in Supplementary Table S6, and the full LASSO selection process, including cross-validation curves and coefficient paths, is shown in Supplementary Figure S4.

### Multivariate logistic regression analysis

**Figure 3:**
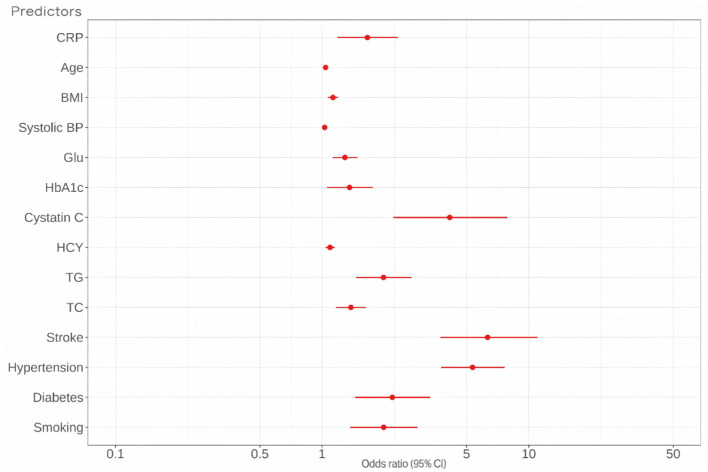
Forest plot of multivariate logistic regression analysis. The odds ratios (ORs) and 95% confidence intervals (CIs) of the 10 independent predictors associated with CSVD. All variables were screened through stepwise AIC method in logistic regression. CI intervals not crossing 1.0 indicated statistically significant associations.

After LASSO-based feature selection, stepwise multivariable logistic regression was performed to further refine predictors associated with CSVD occurrence using the stepAIC procedure, resulting in 14 retained factors. The overall model demonstrated good fit (AIC = 1,006; BIC = 1,082; Nagelkerke R^2^ > 0.50). The odds ratios (ORs) and 95% confidence intervals (CIs) of the selected predictors are summarized in Table 2 and Figure 3. Stroke (OR = 6.323, 95% CI: 3.69–10.38), hypertension (OR = 5.346, 95% CI: 3.76–7.60), and cystatin C (OR = 4.14, 95% CI: 2.20–7.82) showed statistically significant associations with CSVD. All variance inflation factors (VIFs) ranged from 1.01 to 1.10, indicating good independence among the 14 covariates and no evidence of multicollinearity; therefore, the regression coefficient estimates were considered reliable (see Supplementary Table S7).

**Table 2:** Multivariate logistic regression analysis of variables related to CSVD.

Characteristics	β	SE	HR	CI	Z	*P*
Systolic_BP	0.029	0.00615	1.029	1.029 (1.017–1.042)	4.663	<0.001
Age	0.039	0.01051	1.039	1.039 (1.018–1.061)	3.683	<0.001
HCY	0.088	0.02465	1.092	1.092 (1.041–1.146)	3.583	<0.001
BMI	0.121	0.02857	1.129	1.129 (1.067–1.194)	4.244	<0.001
Glu	0.254	0.06944	1.289	1.289 (1.125–1.477)	3.658	<0.001
TC	0.32	0.08464	1.378	1.378 (1.167–1.626)	3.786	<0.001
HbA1c	0.307	0.12944	1.359	1.359 (1.054–1.751)	2.369	<0.05
TG	0.684	0.15681	1.982	1.982 (1.458–2.695)	4.363	<0.001
CRP	0.505	0.17092	1.657	1.657 (1.186–2.317)	2.956	<0.01
Hypertension	1.676	0.17979	5.346	5.346 (3.758–7.604)	9.324	<0.001
Smoking	0.686	0.19063	1.986	1.986 (1.367–2.886)	3.6	<0.001
Diabetes	0.783	0.21257	2.187	2.187 (1.442–3.318)	3.682	<0.001
Stroke	1.844	0.27477	6.323	6.323 (3.690–10.834)	6.712	<0.001
Cystatin_C	1.421	0.32393	4.143	4.143 (2.196–7.818)	4.388	<0.001

### Optimization of the prediction model using recursive feature elimination

**Figure 4:**
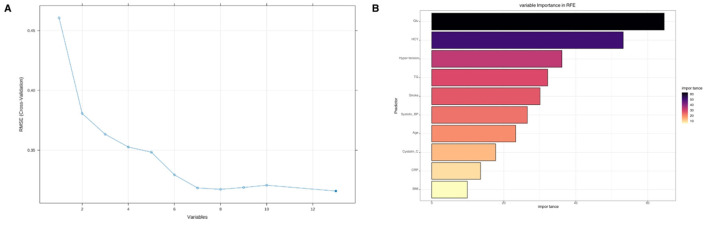
Feature selection using recursive feature elimination in the CSVD prediction model. **(A)** Variation in cross-validated prediction error with increasing numbers of selected features. **(B)** Importance ranking of the top predictors retained by the RFE procedure.

To further optimize the rational clinical application of the model, this study employed a random forest algorithm combined with five-fold cross-validation to systematically screen candidate predictive variables. As shown in Figure 4A, model performance reached its optimum when 10 variables were retained. In addition, random forest–based feature selection identified the 10 most influential core predictors, including blood glucose, history of hypertension, systolic blood pressure, age, triglycerides, history of stroke, cystatin C, C-reactive protein, homocysteine (HCY), and body mass index (BMI). The ranking of feature importance for these variables is presented in Figure 4B, and the corresponding model performance metrics are summarized in Supplementary Table S8.

### Comparative performance of machine learning Models

**Figure 5:**
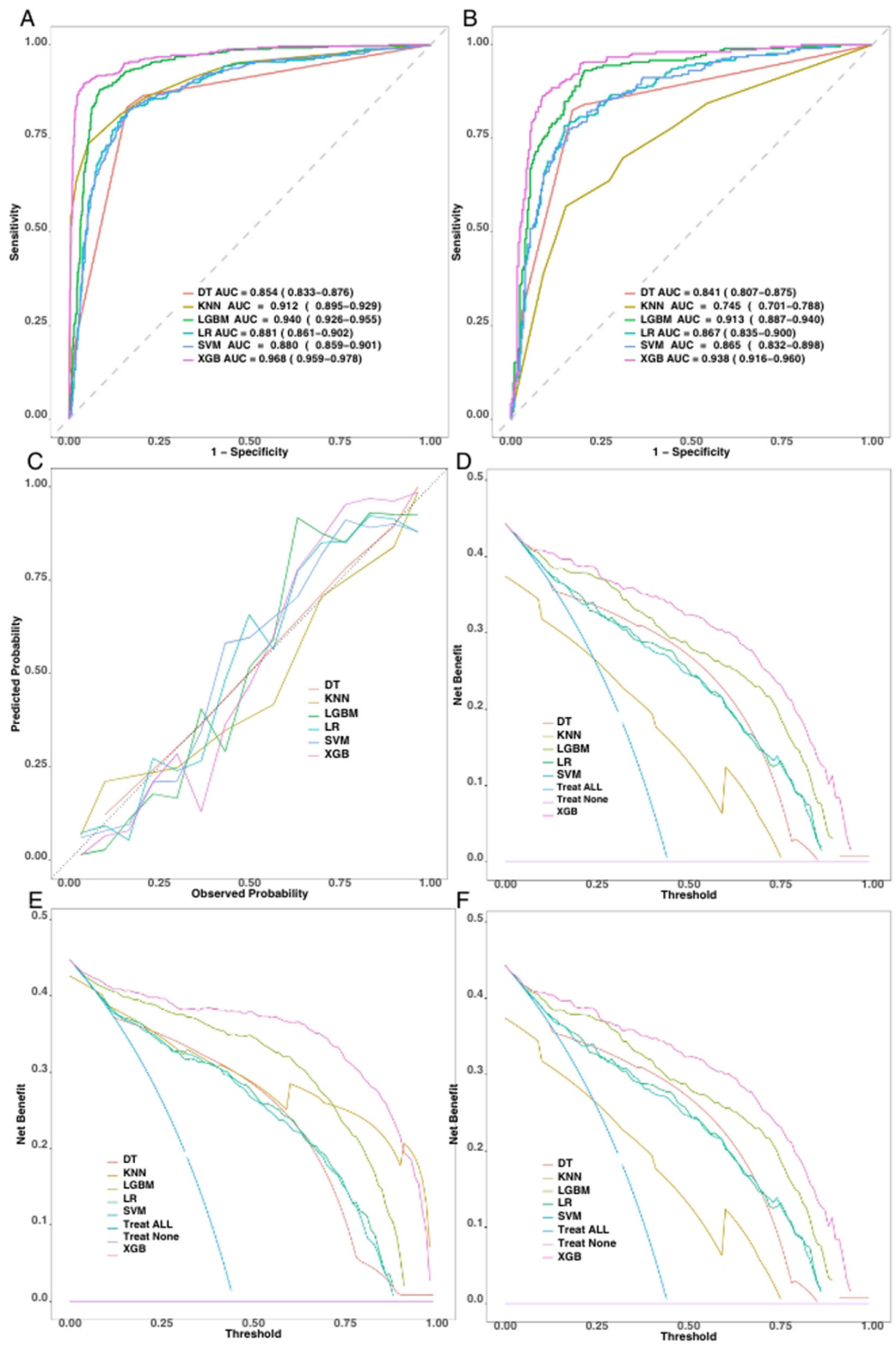
Comprehensive performance evaluation of six machine learning models. **(A)** ROC curves of all six models in the training set. **(B)** ROC curves in the validation set. **(C)** Calibration curves in the training set. **(D)** Calibration curves in the validation set. **(E)** Decision curve analysis (DCA) of models in the training set. **(F)** DCA in the validation set. Note: The models include logistic regression (LR), k-nearest neighbors (KNN), decision tree (DT), support vector machine (SVM), eXtreme Gradient Boosting (XGBoost), and LightGBM (LGBM). XGBoost consistently demonstrated superior performance in discrimination, calibration, and net benefit.

To further enhance discriminative performance and generalizability, six machine learning (ML) algorithms were developed based on the previously selected variables: decision tree (DT), k-nearest neighbors (KNN), logistic regression (LR), Light Gradient Boosting Machine with histogram-based implementation (LGBM), extreme gradient boosting (XGBoost), and support vector machine (SVM). Key hyperparameters and model characteristics are summarized in Table 3. Discriminative performance for each model was evaluated using receiver operating characteristic (ROC) analysis. As illustrated in Figures 5A, B, XGBoost achieved the highest area under the ROC curve (AUC) in both the training and validation cohorts, followed by LGBM. Detailed AUC comparisons are provided in Supplementary Tables S9 (training cohort), S10 (validation cohort), and S11 (combined dataset).In addition to discrimination, model calibration was assessed using calibration curves and Brier scores. As shown in Figures 5C, D, XGBoost and LR demonstrated the closest agreement between predicted and observed probabilities. Quantitative evaluation using Brier scores further supported these findings, with XGBoost yielding the lowest scores in both the training and validation cohorts (Supplementary Tables S12 and S13). Clinical utility was examined through decision curve analysis (DCA). As depicted in Figures 5E, F, XGBoost and LGBM consistently provided the greatest net benefit across a wide range of threshold probabilities.To evaluate model performance in the context of class imbalance, precision–recall (PR) curves were constructed. As shown in Supplementary Figure S5A–B, XGBoost and LGBM achieved the highest areas under the PR curve (AUPRC), with corresponding values summarized in Supplementary Tables S14, S15. Overall, XGBoost outperformed the remaining models across most evaluation metrics. This advantage was further visualized in standardized performance heatmaps (Supplementary Figures 6A–B), where XGBoost consistently ranked highest, followed by LGBM. Bar plots comparing AUC values across models (Supplementary Figures S7A–B) provided an intuitive representation of XGBoost's superior performance across all datasets.Collectively, these results indicate that among the six candidate models, XGBoost exhibited the most favorable and consistent performance across all key evaluation domains—including discrimination, calibration, clinical utility, and precision—and was therefore identified as the optimal model for subsequent interpretation and deployment. Accordingly, XGBoost was selected as the final predictive model. A complete panel of discrimination, calibration and reclassification metrics—AUC, AUPRC, accuracy, sensitivity, specificity, PPV, NPV, F1 score, Matthews correlation coefficient, Cohen's κ, Brier score, log-loss and the Youden index—for all six models in both cohorts is tabulated in Supplementary Tables S16 (training) and S17 (validation) and visualized as a metric matrix therein. On the validation cohort XGBoost attained the highest AUC (0.938) and an overall accuracy of 88.0% (sensitivity 80.9%, specificity 93.8%, F1 0.86, MCC 0.76), exceeding logistic regression by 5.4 AUC points and 6.9 accuracy points. Stratified ten-fold cross-validation reproduced this ordering (XGBoost mean AUC 0.934 ± 0.016, LGBM 0.939 ± 0.014, SVM 0.925 ± 0.024), with narrow standard deviations indicating stable, non-overfitted generalization (Supplementary Table S18, Supplementary Figure S8). Importantly, this accuracy was achieved at negligible computational cost: XGBoost trained in approximately 30 ms with a peak memory footprint below 0.05 MB and a serialized model size of only 65 KB, the most memory-efficient configuration among the gradient-boosting candidates (Supplementary Table S19, Supplementary Figure S9). Beyond these aggregate metrics, several complementary analyses further characterized the selected model: the 10 retained predictors exhibited negligible mutual correlation (all |ρ| ≤ 0.22, all variance-inflation factors < 1.2; Supplementary Figure S10), confirming the low multicollinearity implied by their variance-inflation factors; a threshold-sweep analysis showed that sensitivity, specificity and F1 remained stable across a broad range of cut-offs, with the Youden-optimal threshold (0.44) lying close to the default 0.50 (Supplementary Figure S11); the XGBoost-predicted probabilities were clearly separated between patients with and without CSVD in both cohorts (Supplementary Figure S12); and 1,000-resample bootstrapping yielded a validation-cohort AUC of 0.937 (95% CI 0.915–0.957), whose interval overlapped only with that of LGBM (Supplementary Figure S13). Finally, learning curves for the top-performing models (Supplementary Figure S14) demonstrated progressive convergence of the training and cross-validation AUC with increasing sample size and a narrowing train–validation gap, providing direct visual evidence that the final models are well regularized rather than overfitted.

**Table 3:** Comparison of hyperparameters and performance indicators for six machine learning models.

Model	dfclass	Sensitivity	Specificity	Pos pred value	Neg pred value	Precision	Recall	F1
DT	dev	0.81	0.86	0.83	0.84	0.83	0.81	0.82
KNN	dev	0.73	0.95	0.92	0.81	0.92	0.73	0.82
LGBM	dev	0.88	0.91	0.89	0.90	0.89	0.88	0.89
LR	dev	0.80	0.85	0.82	0.84	0.82	0.80	0.81
SVM	dev	0.83	0.83	0.80	0.80	0.80	0.83	0.81
XGB	dev	0.90	0.96	0.95	0.92	0.95	0.90	0.92
DT	vad	0.79	0.86	0.83	0.83	0.83	0.79	0.81
KNN	vad	0.57	0.85	0.75	0.71	0.75	0.57	0.65
LGBM	vad	0.79	0.88	0.84	0.84	0.84	0.79	0.81
LR	vad	0.79	0.83	0.79	0.83	0.79	0.79	0.79
SVM	vad	0.79	0.80	0.76	0.83	0.76	0.79	0.78
XGB	vad	0.82	0.92	0.90	0.87	0.90	0.82	0.86

Includes sensitivity, specificity, precision, recall, F1-score, prevalence, detection rate, and balanced accuracy for each model.

### Model interpretability and SHAP-based explanation of the XGBoost model

**Figure 6:**
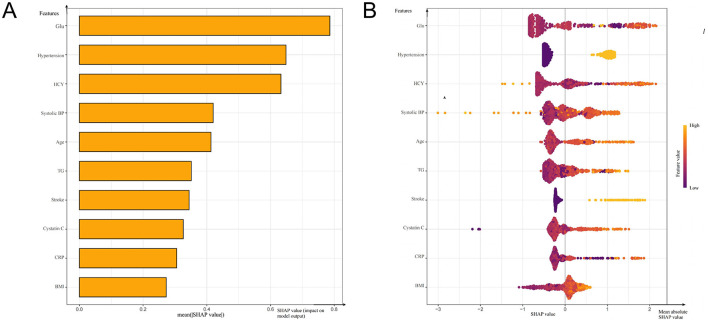
Global interpretability of the XGBoost model using SHAP values **(A)** SHAP summary bar plot showing average absolute SHAP values for the top features. **(B)** SHAP beeswarm plot visualizing the distribution and directionality of SHAP values across individual samples. Warmer colors indicate higher feature values.

To optimize model performance and mitigate overfitting, extensive hyperparameter tuning was conducted using test log loss as the optimization criterion, incorporating grid search and early stopping strategies. The final selected parameters were as follows: booster = “gbtree”, eta = 0.3, gamma = 0.001, max_depth = 2, subsample = 0.7, colsample_bytree = 0.4, and objective = “binary:logistic”. The model achieved optimal performance at the 71st boosting iteration, with a minimum test log loss of 0.324 (Table 4). To elucidate the internal decision-making process of the model and enhance clinical interpretability, Shapley Additive exPlanations (SHAP) were applied at both global and individual levels. Feature importance was first ranked using XGBoost's built-in metrics—gain, cover, and frequency. Across all three measures, a history of stroke and hypertension consistently ranked among the top predictors, indicating their central role in driving model predictions. Cystatin C also maintained a high ranking across importance metrics, highlighting the stable predictive contribution of renal function status. Age, homocysteine (HCY), and inflammation- and metabolism-related variables served as complementary features, primarily contributing to further stratification within different risk tiers (Supplementary Figure S15A–C).The SHAP summary plot provided a high-resolution overview of global feature effects. The bar plot (Figure 6A) demonstrated that glucose (Glu) exerted the greatest mean impact on model output, followed by hypertension, HCY, systolic blood pressure (Systolic_BP), age, and triglycerides (TG). Stroke history, Cystatin C, and C-reactive protein (CRP) showed moderately lower importance, whereas body mass index (BMI) contributed the least. The accompanying beeswarm plot (Figure 6B) illustrated the directional relationships, indicating that higher levels of Glu, hypertension, HCY, systolic blood pressure, age, and TG were strongly associated with increased predicted risk.To explore nonlinear relationships and feature interactions, SHAP dependence analyses were performed for all predictors (Supplementary Figure S16). Elevated glucose levels were found to markedly shift predictions toward higher-risk categories. At the individual level, force plots and waterfall plots were generated to illustrate local explanations. Supplementary Figure S17A–B depicts representative negative samples, whereas Supplementary Figure S17C–D shows positive samples. In negative cases (Supplementary Figure S17A–B), glucose and a history of hypertension dominated the prediction, followed by age and systolic blood pressure, suggesting that metabolic abnormalities and cumulative blood pressure burden constitute fundamental determinants of outcome. Predictions were not driven by a single variable but rather by the combined influence of multisystem risk factors, consistent with low-risk classification. In contrast, positive cases (Supplementary Figure S17C–D) demonstrated a stepwise accumulation of feature contributions from baseline to the final prediction, with strong additive effects of HCY, hypertension, glucose, and systolic blood pressure pushing the predicted probability beyond the decision threshold. Waterfall plots further decomposed the contribution of each feature, enhancing transparency. Confusion matrices derived from the training and validation cohorts (Supplementary Figure S18A–B) demonstrated high classification accuracy and balanced sensitivity and specificity, underscoring the clinical applicability of the model for risk stratification.

**Table 4:** Hyperparameter configuration and tuning summary for the final XGBoost model.

Parameter	Value	Description
booster	gbtree	Type of base learner
eta	0.3	Learning rate
gamma	0.001	Minimum loss reduction to make a further partition
max_depth	2	Maximum depth of a tree
subsample	0.7	Subsample ratio of training instances
colsample_bytree	0.4	Subsample ratio of columns when constructing each tree
objective	binary:logistic	Logistic regression for binary classification
early_stopping_rounds	200	Number of rounds with no improvement to trigger stopping
best_iteration	71	Iteration with lowest test log-loss
best_test_logloss	0.324	Minimum log-loss on validation set

### Nomogram construction and comprehensive evaluation

**Figure 7:**
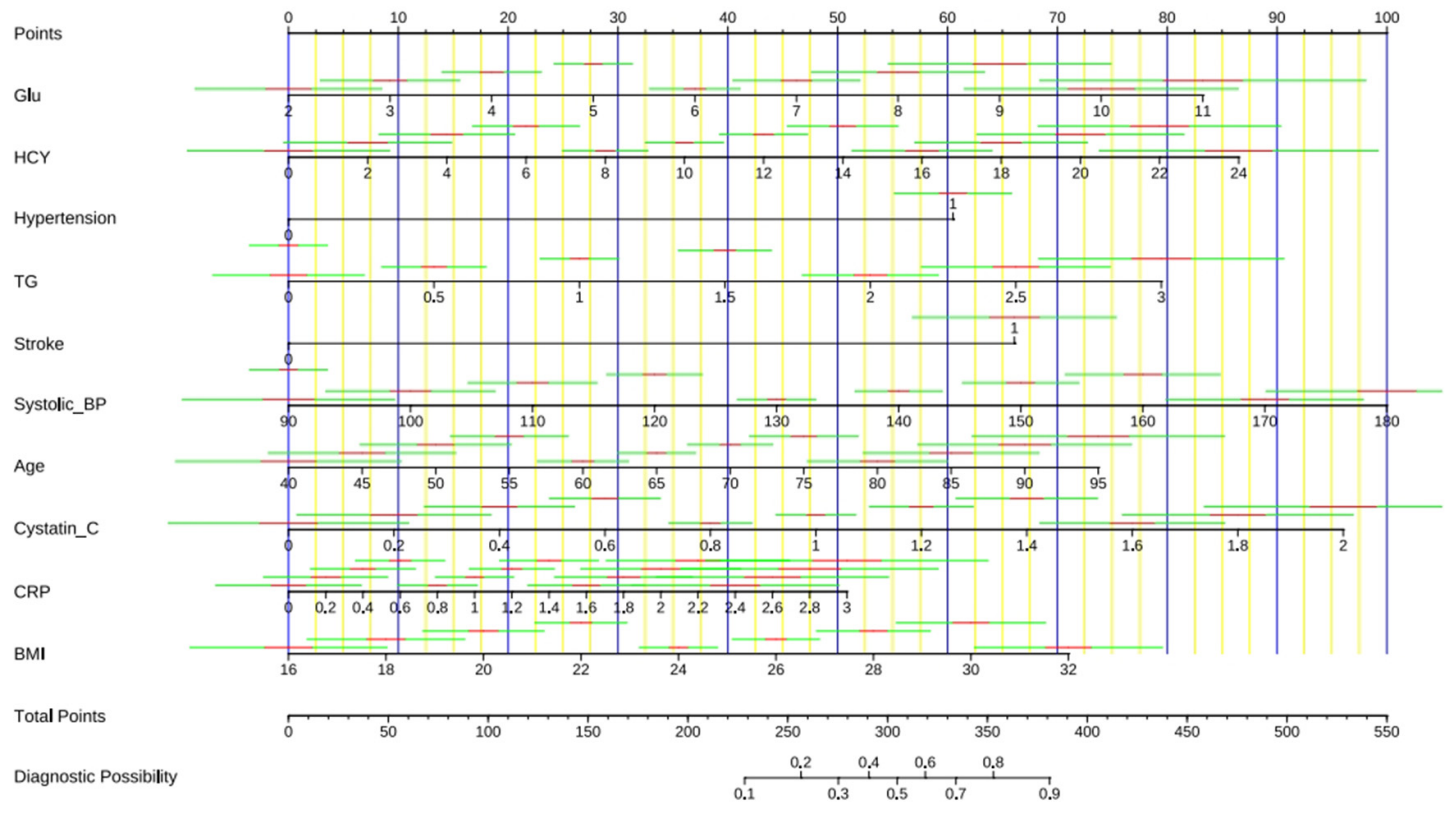
Nomogram based on the 10 most important features from the XGBoost model. Each variable contributes to a total nomoScore, which maps to a predicted probability of adverse outcome.

To facilitate individualized risk prediction and bedside application, a clinically interpretable nomogram was constructed by integrating the top ten predictors identified by the SHAP-guided XGBoost model (Figure 7). Each variable was assigned a weighted score proportional to its contribution, and the cumulative nomogram score corresponded to the predicted probability of adverse outcomes. This graphical tool provides clinicians with a rapid quantitative framework for individualized risk assessment.To rigorously evaluate nomogram performance, a two-tier validation strategy was employed. First, the reliability of the nomogram's probability outputs was assessed. ROC curve analysis in both the training and validation cohorts demonstrated high AUC values, confirming robust discriminative capability (Supplementary Figure S19). Complementary decision curve analysis (DCA) showed superior net benefit across a broad range of threshold probabilities, supporting its clinical utility in real-world settings (Supplementary Figure S20). Diagnostic performance of the prediction model using both continuous probabilities and binarized outcomes in the training and validation cohorts is detailed in Supplementary Table S20, indicating good consistency and clinical applicability. Second, patient-specific nomogram scores (nomoScore) were derived and evaluated for prognostic and diagnostic value. Quartile-based logistic regression analysis revealed a stepwise increase in risk across nomoScore categories: compared with Q1, the odds ratios for Q2, Q3, and Q4 were 2.78, 19.31, and 91.47, respectively (Supplementary Table S21), confirming a strong association between higher nomoScore and CSVD. A forest plot (Supplementary Figure S21) further demonstrated that increasing nomoScore was associated with progressively elevated CSVD risk. Diagnostic performance at the optimal cutoff value was quantified using sensitivity, specificity, accuracy, and the Youden index, with detailed results provided in Supplementary Table S22. Stratified analyses revealed marked differences between patients with and without adverse outcomes, as evidenced by distinct intergroup distributions of nomoScore (Supplementary Figure S22) and risk-stratified bar plots (Supplementary Figure S23), further reinforcing its discriminative capacity. Taken together, these multidimensional validation results indicate that nomoScore, as a nomogram-derived individualized decision and risk stratification biomarker, demonstrates both strong clinical relevance and statistical robustness.

## Discussion

In this retrospective observational study, we developed and validated a machine learning–based predictive model to identify risk factors associated with cerebral small vessel disease (CSVD). Using a rigorously curated cohort of 1,640 patients, 10 independent predictors were identified through least absolute shrinkage and selection operator (LASSO) regression followed by multivariable logistic regression analysis. These predictors included admission glucose level, history of hypertension, systolic blood pressure, age, triglycerides, prior stroke, cystatin C, C-reactive protein, homocysteine, and body mass index (BMI). Among six competing machine learning models, the XGBoost algorithm demonstrated the most favorable performance in terms of discrimination, calibration, and clinical utility, and was therefore selected as the final predictive model. To enhance interpretability and facilitate clinical application, a nomogram incorporating these 10 key variables was constructed. The nomogram-derived score was further validated using logistic regression analysis, diagnostic performance metrics, and stratified visualizations. Collectively, this study provides a robust, interpretable, and clinically actionable tool to support early risk stratification and personalized intervention in patients with CSVD.

Among the identified predictors, admission glucose level emerged as the most influential independent risk factor. Previous studies have demonstrated that diabetes-related cerebrovascular endothelial dysfunction, blood–brain barrier (BBB) disruption, chronic inflammation, and insulin resistance play critical roles in the initiation and progression of CSVD (18, 19). Elevated glucose levels are associated with an increased risk of CSVD, potentially due to excessive intracellular nutrient influx under hyperglycemic conditions, which induces cellular activation or suppression and promotes oxidative stress through multiple metabolic byproducts. These processes activate inflammatory pathways, leading to apoptosis and cellular senescence, while damaged cells release inflammatory mediators that increase BBB permeability and impair cerebral microvasculature integrity (20). Hypertension has been consistently shown to be closely associated with CSVD, contributing to microvascular structural remodeling and impaired cerebrovascular autoregulation through multiple mechanisms (21). Experimental and clinical studies indicate that hypertension suppresses nitric oxide production, disrupts endothelial function, and induces neurovascular injury and BBB dysfunction, ultimately resulting in small vessel damage (22). Interventional studies in patients with lacunar infarction have demonstrated that antihypertensive therapy can improve BBB integrity, promote vasodilation, and attenuate inflammatory responses, thereby slowing CSVD progression (23). Furthermore, a meta-analysis published in 2020 reported that intensive blood pressure control significantly delayed the progression of white matter hyperintensities (WMHs), with the magnitude of benefit positively correlated with the degree of blood pressure reduction (24).

Serum homocysteine (Hcy), a key metabolite in methionine metabolism, exerts direct toxic effects on vascular endothelial cells, accelerating atherosclerotic and microvascular pathological processes (25). Prior studies have suggested a potential association between elevated Hcy levels and CSVD (26), with small vessels appearing more susceptible to Hcy-induced injury than large vessels (27). Increased Hcy levels promote oxidative stress, platelet activation, thrombogenesis, and inflammatory cascades, leading to repeated endothelial damage. Additionally, Hcy induces matrix metalloproteinase-9 activation, disrupting BBB integrity, and accelerates endothelial injury, arteriolosclerosis, smooth muscle cell proliferation, and inflammatory responses, ultimately exacerbating white matter demyelination (28, 29).

Advancing age represents a fundamental determinant of CSVD. With aging, cerebral small vessels exhibit reduced compliance and elasticity due to fragmentation of the elastic lamina, which predisposes to arteriolosclerosis and white matter demyelination (30). Both inflammation and aging have been recognized as central contributors to CSVD pathophysiology (31, 32), with aging-related oxidative stress serving as a key driver of endothelial dysfunction (33, 34). Consistent with these findings, our study demonstrated a progressive increase in CSVD prevalence among individuals older than 60 years.

Dyslipidemia constitutes another important risk factor for CSVD (35), as it compromises cerebrovascular integrity and increases the risk of cognitive impairment. Lipid metabolism disorders can disrupt BBB structure and function, and BBB dysfunction itself represents a critical pathological mechanism underlying CSVD (36). Triglyceride (TG) levels have been linked to the development of WMHs and cerebral microbleeds (CMBs) (37, 38) and are closely correlated with inflammatory markers such as C-reactive protein (CRP) (39). In turn, CRP and related inflammatory markers have been implicated in the formation of enlarged perivascular spaces (EPVS) (40, 41). Elevated TG levels may increase vascular wall and BBB permeability through inflammation-mediated pathways, thereby contributing to EPVS formation. Moreover, TG has been associated with BBB dysfunction (37). Hyperuricemia, which is frequently accompanied by elevated TG levels, has also been linked to CSVD, potentially through its effects on hypertension and endothelial dysfunction (42, 43).

CSVD has been shown to increase the incidence of ischemic stroke, exacerbate treatment-related complications, worsen prognosis, and elevate the risk of stroke recurrence (44). Emerging evidence suggests that newly formed infarcts may evolve into chronic CSVD markers (45), and that greater WMH severity is associated with poorer neurological outcomes 3 months after acute ischemic stroke (46). A large meta-analysis involving 20,322 patients with acute ischemic stroke or transient ischemic attack demonstrated that CMBs significantly increased the risk of hemorrhagic complications, thereby adversely affecting clinical outcomes (47).

Cystatin C (CysC), a ubiquitous cysteine protease inhibitor present in multiple tissues and body fluids, plays a role in intracellular and extracellular protein degradation (48). Elevated serum CysC levels have been significantly associated with cognitive impairment, particularly in elderly populations (49). A positive correlation between serum CysC levels and cognitive dysfunction in patients with CSVD has also been reported (50), suggesting that CysC may serve as a potential biomarker for early identification of cognitive decline in CSVD.

Several studies have identified CSVD as a key contributor to recurrent stroke risk (51), enhancing the efficiency of risk stratification for stroke recurrence (52). Elsaid et al. (53) demonstrated that cerebral microbleeds are among the strongest predictors in models forecasting hemorrhagic transformation after stroke. Body mass index (BMI) has been positively associated with the risk of ischemic stroke (54). Compared with individuals with lower BMI, patients with higher BMI are more likely to experience lacunar infarction, whereas cardioembolic stroke predominates among those with lower BMI (55). Although evidence linking BMI to CSVD remains limited, our findings indicate a positive association between BMI and CSVD prevalence.

Our results compare favorably with the small but growing body of machine-learning work on CSVD and adjacent cerebrovascular phenotypes (Supplementary Table S23). Conventional clinical-laboratory nomograms for white-matter hyperintensities have reported validation AUCs of roughly 0.76–0.78 (13, 14, 56), and an explainable stacking ensemble trained on 23 CSVD variables achieved an AUC of 0.881 with a Brier score of 0.127 (15). Imaging-based pipelines can reach higher discrimination—multimodal-MRI XGBoost models report AUCs up to 0.92–0.96 (50) and radiomics-augmented nomograms up to 0.94 (14)—but they depend on advanced sequences and segmentation—or, for diffusion-based dementia prediction, specialized diffusion-tensor acquisition (57)—that are unavailable in most primary-care and emergency settings. Against this backdrop, the present model attained a validation AUC of 0.938 and an accuracy of 88.0% using only ten routinely measured, inexpensive variables, matching imaging-dependent approaches while preserving full SHAP-level interpretability and bedside deployability. A community-based interpretable CSVD screening tool built on a comparable algorithm set (LR, SVM, RF, XGBoost, LightGBM) likewise identified gradient boosting as the optimal learner (49), and tree-based boosting has consistently outperformed linear and kernel models in haemorrhagic-stroke outcome prediction (16, 17) and ischaemic-stroke functional prognosis (14), reinforcing the external coherence of our findings.

This study has several notable strengths. First, it represents one of the few investigations to develop and validate a clinically interpretable, machine learning–driven prediction model specifically for patients with CSVD. Second, rigorous feature selection using LASSO regression combined with AIC-based multivariable regression ensured optimal model parsimony while minimizing overfitting. Third, SHAP analysis was employed to enhance model transparency and clinician interpretability, facilitating bedside application. Fourth, the constructed nomogram translated complex machine learning outputs into a practical tool for individualized prognostic assessment and therapeutic decision-making.Nevertheless, several limitations warrant consideration. First, this was a single-center retrospective study, which may limit generalizability. Although internal validation was performed, future multicenter studies with external validation are needed. Second, imaging biomarkers were not incorporated; high-field MRI data could further improve risk prediction accuracy. Third, longitudinal follow-up and post-discharge rehabilitation data were not included, which may have enriched prognostic modeling. Fourth, although ten-fold cross-validation, calibration testing and DCA argue against substantial overfitting, the absence of a geographically independent external cohort means the reported metrics may still be optimistic; the tree-based models also showed a modest train-to-test drop in sensitivity (e.g., XGBoost 0.87 to 0.81), indicating residual variance that a larger multi-site sample could reduce. Fifth, a single 0.5 probability threshold was used for the confusion-matrix metrics, whereas clinical deployment may warrant cost-sensitive or prevalence-adjusted cut-offs. Looking forward, our future work will (i) pursue prospective multicentre external validation across hospitals of differing tiers and ethnic case-mix, (ii) integrate quantitative neuroimaging markers (white-matter-hyperintensity volume, lacune and microbleed counts) and longitudinal outcomes to extend the model from cross-sectional risk identification to dynamic prognosis, (iii) explore federated and continually updated learning to protect privacy while improving transportability, and (iv) embed the nomogram and its SHAP explanations into an electronic-health-record decision-support interface for real-world impact assessment.

In conclusion, our findings demonstrate that a machine learning–derived nomogram integrating glucose, hypertension, homocysteine, systolic blood pressure, age, triglycerides, and related factors can accurately predict adverse outcomes in patients with CSVD. By capturing multidimensional clinical information and translating it into an actionable clinical tool, this model offers meaningful support for early risk stratification and personalized management. Future research should focus on real-world validation, dynamic outcome prediction, and integration with treatment algorithms to optimize care for this high-risk population.

### Implications of the study

This study has important implications for clinical practice, technical translation, and healthcare governance. Clinically, the explainable XGBoost model and accompanying nomogram may help physicians identify patients at higher risk of cerebral small vessel disease using routinely available variables, thereby supporting earlier recognition, individualized risk stratification, and more targeted preventive or therapeutic management. From a technical standpoint, the integration of XGBoost with SHAP and nomogram-based visualization offers an interpretable framework for clinical risk prediction and provides a feasible basis for future implementation within routine decision-support workflows. More broadly, this work also has policy relevance in the context of artificial intelligence in medicine, where transparency and interpretability are essential for clinician trust, regulatory oversight, and responsible adoption of machine learning tools in real-world healthcare settings.

## Conclusion

In this study, we developed and validated an interpretable XGBoost-based prediction model for cerebral small vessel disease using routinely collected clinical data. Among six machine learning algorithms, XGBoost demonstrated the most favorable overall performance in terms of discrimination, calibration, and clinical utility. The integration of SHAP analysis further improved model transparency by identifying the relative contribution and direction of key predictors, while the nomogram translated the model output into a more accessible tool for individualized risk estimation. Clinically, the identified predictors—particularly glucose, hypertension, homocysteine, systolic blood pressure, age, and triglycerides—may help support earlier recognition of patients at elevated risk. Taken together, these findings suggest that explainable machine learning may provide meaningful support for precision risk stratification in CSVD. Nevertheless, prospective multicenter studies and external validation are still required before routine clinical implementation. Future work will extend the present cross-sectional framework toward prospective multicentre external validation, incorporation of quantitative neuroimaging and longitudinal outcomes, privacy-preserving federated updating, and prospective evaluation of the nomogram once embedded within electronic-health-record decision support.

## Data Availability

The raw data supporting the conclusions of this article will be made available by the authors, without undue reservation.
