# Supplementary material for: Explainable XGBoost model and nomogram for risk factor identification and risk prediction in cerebral small vessel disease: a machine learning-based retrospective cohort study
Source: Front Neurol. 2026 Jul 24;17:1786279. doi: 10.3389/fneur.2026.1786279 (PMC13447219; doi:10.3389/fneur.2026.1786279)
Supplement: Supplementary file 1 [file Supplementary_file_1.DOCX]

**Supplementary Figure S1. End-to-end analytical workflow of the study.**Schematic of the complete modelling pipeline, from assembly of the 1,640-patient cohort and application of inclusion/exclusion criteria, through the stratified 7:3 training–validation split and the three-stage feature-selection cascade (univariable screening → LASSO with 10-fold cross-validation → stepwise multivariable logistic regression with variance-inflation-factor check → random-forest recursive feature elimination yielding the final ten predictors), to the training and head-to-head comparison of six supervised algorithms under stratified 10-fold cross-validation. Models were evaluated for discrimination (AUC/ROC/precision–recall), calibration (Brier score, Hosmer–Lemeshow) and clinical utility (decision-curve analysis); the validation cohort (blue path) was reserved exclusively for held-out evaluation. The best-performing model (XGBoost) was subsequently interpreted with SHAP and translated into a bedside nomogram and nomoScore.

**Supplementary Figure S2. Schematic mind-map of the explainable XGBoost modelling pipeline.**Conceptual overview organising the methodology into six themed branches radiating from the central pipeline: (1) data and cohort (1,640 patients, no missing data, stratified 7:3 split, 23 candidate predictors); (2) feature selection (univariable screening, LASSO with 10-fold cross-validation, stepwise AIC with variance-inflation-factor check, and recursive feature elimination yielding ten predictors); (3) model development (six algorithms, grid-search tuning, stratified 10-fold cross-validation); (4) model selection by discrimination, calibration and decision-curve criteria, identifying XGBoost; (5) SHAP-based global and local interpretation; and (6) clinical translation into a weighted nomogram and nomoScore with bootstrap and learning-curve validation.

**Supplementary Figure S3. Architecture of the selected XGBoost classifier.**The ten-dimensional input feature vector is passed to an additive ensemble of K = 71 shallow regression trees (maximum depth = 2). Each successive tree is fitted to the negative gradient (pseudo-residual) of the current ensemble; the weighted sum of all tree outputs is mapped through a logistic link to yield the predicted probability of cerebral small vessel disease.

**Supplementary Figure S4. Coefficient distribution of selected variables in Lasso regression**

**Supplementary Figure S5. Precision-Recall (PR) curves and radar-based comparison of six machine learning models.** (A) PR curves of all models in the training set. (B) PR curves in the validation set.

**Supplementary Figure S6. Heatmaps of performance metrics for different machine learning models.** (A) Heatmaps of standardized AUC, PR-AUC, F1 score, and Brier score in the training set. (B) Heatmaps of the same metrics in the validation set.

**Supplementary Figure S7. Comparison of AUC values for six models in bar charts.** (A) AUC values of each model in the training set. (B) AUC values in the validation set.

**Supplementary Figure S8. Ten-fold cross-validated discrimination across the six models.**Distribution of the area under the ROC curve (AUC) across ten stratified cross-validation folds for each model. Boxes show the interquartile range, the central line the median, the white diamond the mean, and grey points the individual fold values; the mean ± SD is annotated below each box. The narrow dispersions (AUC SD ≤ 0.024 for every model) confirm stable generalization and argue against overfitting. Gradient-boosting learners (LGBM and XGBoost) and SVM occupy the top performance tier.

**Supplementary Figure S9. Computational efficiency of the six models.**(A) Wall-clock training time (ms) for fitting each model on the 1,148-patient training cohort and (B) peak training-memory footprint (MB). All models trained in well under 0.1 s and required ≤ 1.5 MB of peak memory. The selected XGBoost model combined high accuracy with the smallest gradient-boosting memory footprint (< 0.05 MB), demonstrating that state-of-the-art predictive performance is achievable on ordinary CPU-only clinical hardware.

**Supplementary Figure S10 Inter-predictor correlation structure and multicollinearity assessment.** (A) Spearman rank-correlation matrix of the ten predictors retained in the final model and (B) the corresponding variance inflation factors (VIF). All pairwise correlations were weak (maximum |ρ| = 0.22) and every VIF was far below the pre-specified threshold of 3.2 (maximum VIF = 1.15), confirming that the selected predictors are statistically independent and that the multivariable model is free of problematic multicollinearity.

**Supplementary Figure S11. Threshold-dependent performance of the XGBoost model.**Sensitivity, specificity, F1 score, accuracy and Youden’s J index of the final XGBoost model in the validation cohort plotted against the classification threshold. The metrics remained stable over a broad range of cut-offs, and the empirically optimal threshold maximizing Youden’s J (0.44; J = 0.79, dash-dotted line) lay close to the default 0.50 cut-off used for all threshold-dependent metrics in this study, while the curves provide a transparent basis for cost- or prevalence-adjusted re-thresholding in future deployment.

**Supplementary Figure S12. Separation of XGBoost-predicted probabilities by true disease status.**Density histograms of the XGBoost-predicted probability of CSVD, stratified by true disease status, in the (A) training and (B) validation cohorts (dashed line, 0.50 threshold). The predicted-probability distributions of patients with and without CSVD were clearly separated in both cohorts, with limited overlap around the decision boundary, providing an intuitive, sample-level visualisation of the model’s discriminative behaviour that is distinct from the aggregate ROC/PR analyses shown elsewhere.

**Supplementary Figure S13. Bootstrap confidence intervals for validation-cohort AUC.**Mean and 95% confidence interval of the validation-cohort AUC for each model, obtained from 1,000 stratified bootstrap resamples of the held-out cohort; the XGBoost marker is enlarged. XGBoost achieved the highest bootstrap AUC (0.937, 95% CI 0.915–0.957), with an interval that overlapped only that of LGBM (0.935, 95% CI 0.911–0.956) and lay entirely above those of logistic regression, k-nearest neighbours and the decision tree.

**Supplementary Figure S14. Learning curves demonstrating convergence and absence of overfitting.**Training AUC (red) and ten-fold cross-validation AUC (blue) as a function of the training-set size for the three top-performing models (XGBoost, LGBM and SVM); shaded bands denote ± 1 standard deviation across folds. For every model the cross-validation curve rises monotonically and the gap between the training and cross-validation curves narrows as more data are added, with the curves approaching a stable plateau. This convergent behaviour is the canonical signature of a well-regularised, non-overfitted model and complements the cross-validation, calibration and decision-curve analyses reported elsewhere.

**Figure S15. Feature importance ranking based on the XGBoost model:** (A) Importance ranked by gain, indicating each feature's relative contribution to model improvement; (B) Importance ranked by coverage, measuring the number of observed values affected by each feature; (C) Importance ranked by frequency, reflecting the feature's frequency in the model's split tree

**Figure S16. Shap dependency graph of the first four features in the XGBoost model**

**Figure S17. Individualized Shap force plots of representative samples (A–B) Negative prediction cases: The force plots and corresponding waterfall plots demonstrate the minimum and subtractive Shap contributions of all variables, resulting in lower prediction probabilities. (C–D) Positive prediction cases: The force plots and waterfall plots illustrate the additive contributions of relevant factors, leading to higher prediction probabilities.**

**Figure S18.** **Confusion matrix of the XGBoost mode**l (A) Confusion matrix in the training cohort. (B) Confusion matrix in the validation cohort.

**Supplementary Figure S19. ROC curves derived from column-line plot-based prediction probabilities on both training and validation sets.**

**Supplementary Figure S20. Decision Curve Analysis (DCA) of Clinical Utility for Nomogram.**

**Figure S21. Forest plot of the nomoScore subgroup analysis. Stratified by nomoScore score levels; the odds ratio (OR) was consistent across subgroups.**

**Figure S22. Box plot of nomoScore distribution in patients with and without outcomes.**

**Figure S 23. Comparative bar chart of nomoScore levels between positive and negative outcome groups.**
