# Supplementary material for: Explainable XGBoost model and nomogram for risk factor identification and risk prediction in cerebral small vessel disease: a machine learning-based retrospective cohort study
Source: Front Neurol. 2026 Jul 24;17:1786279. doi: 10.3389/fneur.2026.1786279 (PMC13447219; doi:10.3389/fneur.2026.1786279)
Supplement: Supplementary file 2 [file Supplementary_file_2.DOCX]

**Supplementary Table S1. Numerical descriptive statistics and univariable comparison of the 23 candidate predictors between patients with and without CSVD.**

| **Variable (unit)** | **Type** | **Overall (N = 1,640)** | **Non-CSVD (n = 908)** | **CSVD (n = 732)** | **P** |
| --- | --- | --- | --- | --- | --- |
| Age, years | Cont. | 65.00 [61.00, 71.00] | 64.00 [60.00, 68.00] | 68.00 [61.00, 74.00] | <0.001 |
| BMI, kg/m² | Cont. | 24.02 [22.04, 25.96] | 23.31 [21.38, 25.40] | 24.58 [23.12, 26.56] | <0.001 |
| Systolic BP, mmHg | Cont. | 131.00 [122.00, 141.00] | 127.00 [119.00, 136.00] | 137.00 [126.00, 147.00] | <0.001 |
| Diastolic BP, mmHg | Cont. | 79.00 [71.00, 86.00] | 79.00 [72.00, 86.00] | 79.00 [70.00, 87.00] | 0.610 |
| Glucose, mmol/L | Cont. | 5.04 [4.53, 5.68] | 4.95 [4.58, 5.35] | 5.36 [4.36, 6.60] | <0.001 |
| HbA1c, % | Cont. | 5.80 [5.40, 6.20] | 5.70 [5.40, 6.00] | 5.90 [5.40, 6.50] | <0.001 |
| D-dimer, µg/L | Cont. | 104.00 [55.00, 149.25] | 92.00 [50.00, 137.00] | 114.00 [65.75, 163.00] | <0.001 |
| Fibrinogen, g/L | Cont. | 2.75 [2.48, 3.09] | 2.72 [2.48, 2.98] | 2.84 [2.47, 3.21] | <0.001 |
| BUN, mmol/L | Cont. | 5.03 [4.18, 5.61] | 4.99 [4.29, 5.44] | 5.18 [4.10, 5.91] | 0.130 |
| Uric acid, µmol/L | Cont. | 298.00 [247.00, 349.00] | 288.00 [229.75, 348.25] | 308.00 [262.00, 350.00] | <0.001 |
| Creatinine, µmol/L | Cont. | 77.00 [66.00, 86.25] | 74.00 [66.00, 84.00] | 79.00 [68.00, 89.00] | <0.001 |
| Cystatin C, mg/L | Cont. | 0.89 [0.72, 1.07] | 0.87 [0.72, 1.01] | 0.94 [0.73, 1.15] | <0.001 |
| Homocysteine, µmol/L | Cont. | 10.00 [8.00, 12.00] | 9.00 [8.00, 10.00] | 11.00 [8.75, 15.00] | <0.001 |
| CRP, mg/L | Cont. | 0.88 [0.55, 1.21] | 0.83 [0.54, 1.12] | 0.95 [0.55, 1.40] | <0.001 |
| LDL, mmol/L | Cont. | 2.43 [1.98, 2.93] | 2.43 [2.04, 2.83] | 2.44 [1.88, 3.05] | 0.699 |
| Triglycerides, mmol/L | Cont. | 1.03 [0.72, 1.41] | 0.94 [0.66, 1.22] | 1.23 [0.83, 1.67] | <0.001 |
| Total cholesterol, mmol/L | Cont. | 4.00 [3.36, 4.72] | 3.90 [3.31, 4.53] | 4.20 [3.46, 4.97] | <0.001 |
| Sex, male | Bin. | 768 (46.8%) | 437 (48.1%) | 331 (45.2%) | 0.261 |
| Prior stroke | Bin. | 223 (13.6%) | 45 (5.0%) | 178 (24.3%) | <0.001 |
| Hypertension | Bin. | 515 (31.4%) | 117 (12.9%) | 398 (54.4%) | <0.001 |
| Diabetes mellitus | Bin. | 296 (18.0%) | 90 (9.9%) | 206 (28.1%) | <0.001 |
| Smoking | Bin. | 426 (26.0%) | 191 (21.0%) | 235 (32.1%) | <0.001 |
| Alcohol consumption | Bin. | 295 (18.0%) | 145 (16.0%) | 150 (20.5%) | 0.021 |

Continuous variables (Type = Cont.) are summarized as median [Q1, Q3] and compared with the Mann–Whitney U test; binary variables (Type = Bin.) are summarized as the number (percentage) of the coded-positive category (sex: male; others: yes) and compared with the Pearson χ² test. Coding: sex 1 = male/0 = female; all other binary variables 1 = yes/0 = no. All values were computed directly from the study dataset (N = 1,640; 908 non-CSVD, 732 CSVD); no data were imputed as the dataset contained no missing values. The ten variables retained in the final model were glucose, homocysteine, hypertension, triglycerides, prior stroke, systolic blood pressure, age, cystatin C, C-reactive protein and body-mass index. P < 0.05 was considered statistically significant.

**Supplementary Table S2. Sample size and class distribution across the training and validation partitions.**

| **Cohort** | **Total, n** | **CSVD (positive), n (%)** | **Non-CSVD (negative), n (%)** |
| --- | --- | --- | --- |
| Full analytic cohort | 1,640 | 732 (44.6%) | 908 (55.4%) |
| Training cohort (70%) | 1,148 | 512 (44.6%) | 636 (55.4%) |
| Validation cohort (30%) | 492 | 220 (44.7%) | 272 (55.3%) |

The cohort was partitioned by stratified random sampling at a 7:3 ratio, preserving the outcome prevalence across partitions (χ² p = 1.00). The validation cohort was held out and used only for final performance estimation; all feature selection and hyperparameter tuning were performed within the training cohort.

**Supplementary Table S3. Search space and final hyperparameter configuration for the six machine-learning models.**

| **Model** | **Tuned hyperparameters (search grid)** | **Final configuration** |
| --- | --- | --- |
| DT | max_depth {3,5,7,10}; min_samples_leaf {10,20,30}; criterion {gini, entropy} | max_depth = 5; min_samples_leaf = 20; criterion = gini |
| KNN | n_neighbors {5,9,15,21}; weights {uniform, distance}; metric {euclidean, manhattan} | n_neighbors = 15; weights = uniform; metric = euclidean |
| LR | penalty {l1, l2}; C {0.01,0.1,1,10}; solver {lbfgs, liblinear} | penalty = l2; C = 1.0; solver = lbfgs; max_iter = 2000 |
| LGBM | n_estimators {100,200}; num_leaves {15,31}; learning_rate {0.05,0.1}; subsample {0.8,1.0}; colsample_bytree {0.7,1.0} | n_estimators = 120; num_leaves = 15; learning_rate = 0.05; subsample = 0.8; colsample_bytree = 0.7; min_child_samples = 25 |
| **XGBoost** | **eta {0.1,0.3}; max_depth {2,3,4}; subsample {0.7,0.9}; colsample_bytree {0.4,0.7}; gamma {0.001,0.01}; n_estimators (early stopping)** | **booster = gbtree; eta = 0.3; max_depth = 2; subsample = 0.7; colsample_bytree = 0.4; gamma = 0.001; best_iteration = 71** |
| SVM | C {0.1,1,10}; kernel {linear, rbf}; gamma {scale, auto} | C = 1.0; kernel = rbf; gamma = scale; probability = TRUE |

All grids were explored by exhaustive search with stratified 10-fold cross-validation on the training cohort, optimizing the mean cross-entropy (log-loss). For XGBoost the number of boosting rounds was determined by early stopping (patience = 200 rounds). Distance- and margin-based learners (KNN, LR, SVM) were fitted on z-score-standardized features; tree-based learners (DT, LGBM, XGBoost) used the raw feature scale. The XGBoost row (highlighted) denotes the final selected model.

**Supplementary Table S4. Hardware and software environment and associated cost.**

| **Component** | **Specification** | **Cost** |
| --- | --- | --- |
| Processor (CPU) | Standard x86-64 multi-core desktop CPU (no GPU required) | Commodity (≈ US$ 600–900 workstation) |
| Memory (RAM) | 16 GB (peak model memory < 1.5 MB; far below capacity) | Included in workstation |
| Operating system | 64-bit Linux / Windows | Free / pre-installed |
| Programming languages | Python 3 and R 4.2.1 | Free, open-source |
| Core libraries | scikit-learn, XGBoost, LightGBM, SHAP, pandas, NumPy, Matplotlib | Free, open-source (BSD/MIT/Apache) |
| Model training & inference | Full pipeline trains in seconds on CPU | No cloud / GPU cost |
| Deployment | Serialized model (≤ 0.2 MB) + nomogram; runs in a browser or EHR plug-in | Negligible |

The complete analytical and deployment stack relies exclusively on freely licensed, open-source software and runs on a standard CPU-only workstation. No graphics-processing unit, high-performance-computing cluster, or paid cloud service is required. The dominant cost is therefore the one-time purchase of commodity hardware already available in most clinical departments, making the model economically transferable to resource-limited settings.

**Supplementary Table S5. Baseline characteristics of CSVD patients**

|  | [ALL] N=1604 | Non-CSVD N=908 | CSVD N=732 | p.overall |
| --- | --- | --- | --- | --- |
| CRP | 0.88 [0.55;1.21] | 0.83 [0.54;1.12] | 0.95 [0.55;1.40] | <0.001 |
| BUN | 5.03 [4.18;5.61] | 4.99 [4.29;5.44] | 5.18 [4.10;5.91] | 0.130 |
| Drink: |  |  |  | 0.021 |
| No | 1345 (82.01%) | 763 (84.03%) | 582 (79.51%) |  |
| Yes | 295 (17.99%) | 145 (15.97%) | 150 (20.49%) |  |
| Age | 65.00 [61.00;71.00] | 64.00 [60.00;68.00] | 68.00 [61.00;74.00] | <0.001 |
| BMI | 24.00 (2.87) | 23.38 (2.93) | 24.77 (2.61) | <0.001 |
| Systolic_BP | 131.00 [122.00;141.00] | 127.00 [119.00;136.00] | 137.00 [126.00;147.00] | <0.001 |
| Diastolic_BP | 78.76 (10.64) | 78.89 (9.26) | 78.60 (12.13) | 0.586 |
| Glu | 5.04 [4.53;5.68] | 4.95 [4.58;5.35] | 5.36 [4.36;6.60] | <0.001 |
| HbA1c | 5.80 [5.40;6.20] | 5.70 [5.40;6.00] | 5.90 [5.40;6.50] | <0.001 |
| D_dimer | 104.00 [55.00;149.25] | 92.00 [50.00;137.00] | 114.00 [65.75;163.00] | <0.001 |
| FIB | 2.75 [2.48;3.09] | 2.72 [2.48;2.98] | 2.84 [2.47;3.21] | <0.001 |
| Uric_acid | 298.77 (77.76) | 292.51 (85.10) | 306.54 (66.81) | <0.001 |
| Creatinine | 77.00 [66.00;86.25] | 74.00 [66.00;84.00] | 79.00 [68.00;89.00] | <0.001 |
| Cystatin_C | 0.89 [0.72;1.07] | 0.87 [0.72;1.01] | 0.94 [0.73;1.15] | <0.001 |
| HCY | 10.00 [8.00;12.00] | 9.00 [8.00;10.00] | 11.00 [8.75;15.00] | <0.001 |
| LDL | 2.46 (0.73) | 2.44 (0.61) | 2.47 (0.86) | 0.504 |
| TG | 1.03 [0.72;1.41] | 0.94 [0.66;1.22] | 1.23 [0.83;1.67] | <0.001 |
| TC | 4.00 [3.36;4.72] | 3.90 [3.31;4.53] | 4.20 [3.46;4.97] | <0.001 |
| Gender: |  |  |  | 0.261 |
| Male | 872 (53.17%) | 471 (51.87%) | 401 (54.78%) |  |
| Female | 768 (46.83%) | 437 (48.13%) | 331 (45.22%) |  |
| Stroke: |  |  |  | <0.001 |
| No | 1417 (86.40%) | 863 (95.04%) | 554 (75.68%) |  |
| Yes | 223 (13.60%) | 45 (4.96%) | 178 (24.32%) |  |
| Hypertension: |  |  |  | <0.001 |
| No | 1125 (68.60%) | 791 (87.11%) | 334 (45.63%) |  |
| Yes | 515 (31.40%) | 117 (12.89%) | 398 (54.37%) |  |
| Diabetes: |  |  |  | <0.001 |
| No | 1344 (81.95%) | 818 (90.09%) | 526 (71.86%) |  |
| Yes | 296 (18.05%) | 90 (9.91%) | 206 (28.14%) |  |
| Smoke: |  |  |  | <0.001 |
| No | 1214 (74.02%) | 717 (78.96%) | 497 (67.90%) |  |
| Yes | 426 (25.98%) | 191 (21.04%) | 235 (32.10%) |  |

Note: C-reactive protein (CRP), blood urea nitrogen (BUN), body mass index (BMI), glycated hemoglobin (HbA1c), fibrinogen (FIB), uric acid (UA), homocysteine (HCY), low-density lipoprotein (LDL), triglycerides (TG), and total cholesterol (TC). Categorical variables are presented as follows: sex: male, female; hypertension: absent, present; diabetes: absent, present; smoking: absent, present; alcohol consumption: absent, present; stroke: absent, present. Statistical comparison of continuous variables between groups was performed using the Mann-Whitney U test, and categorical variables were analyzed using the χ² test. A P-value <0.05 was considered statistically significant.

**Supplementary Table S6. Variables selected by Lasso regression with λ.min = 0.020 and their coefficients**

| **Variable** | **Coefficient** |
| --- | --- |
| Intercept | –11.847 |
| CRP | 0.260 |
| Age | 0.0244 |
| BMI | 0.0808 |
| Systolic_BP | 0.0222 |
| Glu | 0.142 |
| HbA1c | 0.144 |
| D_dimer | 0.0005 |
| Cystatin_C | 0.739 |
| HCY | 0.0646 |
| TG | 0.538 |
| TC | 0.148 |
| Stroke | 1.302 |
| Hypertension | 1.409 |
| Diabetes | 0.527 |
| Smoking | 0.265 |

Note: This table lists all variables retained using 10-fold cross-validation with the optimal penalty parameter λ.min. Only variables with non-zero coefficients are selected for inclusion in subsequent prediction models.

**Supplementary Table S7. Variance Inflation Factor (VIF) values of retained predictors in the final multivariate logistic regression model**

| **Variable** | **VIF** |
| --- | --- |
| CRP | 1.029232995287261 |
| Age | 1.013771253479729 |
| BMI | 1.011698180930856 |
| Systolic_BP | 1.05092946582652 |
| Glu | 1.03214071083662 |
| HbA1c | 1.016834746036326 |
| Cystatin_C | 1.035737729890109 |
| HCY | 1.039752164951972 |
| TG | 1.032817964146724 |
| TC | 1.096012321974717 |
| Stroke | 1.018326422127386 |
| Hypertension | 1.028876703122013 |
| Diabetes | 1.019050407701533 |
| Smoking | 1.096569319180792 |

Note: All VIF values are below 1.1, indicating no significant multicollinearity among the variables in the final model.

**Supplementary Table S8. Best Model Performance**

| number of variates | RMSE | R² | MAE |
| --- | --- | --- | --- |
| 1 | 0.4607 | 0.2110 | 0.3462 |
| 2 | 0.3807 | 0.4187 | 0.2731 |
| 3 | 0.3633 | 0.4762 | 0.2828 |
| 4 | 0.3527 | 0.5112 | 0.2814 |
| 5 | 0.3484 | 0.5397 | 0.2864 |
| 6 | 0.3295 | 0.5651 | 0.2269 |
| 7 | 0.3185 | 0.5960 | 0.2230 |
| 8 | 0.3174 | 0.6008 | 0.2249 |
| 9 | 0.3189 | 0.5947 | 0.2211 |
| 10 | 0.3208 | 0.5907 | 0.2266 |

**Supplementary Table S9. Area Under the Curve (AUC) values of models in the training set**

|  | DT | LM | XGBOOST | SVM | KNN | LGBM |
| --- | --- | --- | --- | --- | --- | --- |
| DT | 1 | 0.076752 | 5.184513503103842e-33 | 0.008722 | 0.000001 | 7.520320465949527e-11 |
| LM | 0.076752 | 1 | 1.345871285422296e-13 | 0.925195 | 0.025768 | 0.000004 |
| XGBOOST | 5.184513503103842e-33 | 1.345871285422296e-13 | 1 | 1.323906614703991e-25 | 7.68403349982384e-11 | 0.001663 |
| SVM | 0.008722 | 0.925195 | 1.323906614703991e-25 | 1 | 0.002338 | 0.000003 |
| KNN | 0.000001 | 0.025768 | 7.68403349982384e-11 | 0.002338 | 1 | 0.012091 |
| LGBM | 7.520320465949527e-11 | 0.000004 | 0.001663 | 0.000003 | 0.012091 | 1 |

**Supplementary Table S10. Area Under the Curve (AUC) values of models in the validation set**

|  | DT | LM | XGBOOST | SVM | KNN | LGBM |
| --- | --- | --- | --- | --- | --- | --- |
| DT | 1 | 0.264825 | 6.808730773002831e-10 | 0.157225 | 0.000026 | 0.000989 |
| LM | 0.264824 | 1 | 0.000494 | 0.925427 | 0.000010 | 0.032832 |
| XGBOOST | 6.808730773002831e-10 | 0.000494 | 1 | 1.647613720885028e-7 | 8.652486120944639e-20 | 0.159385 |
| SVM | 0.157225 | 0.925427 | 1.647613720885028e-7 | 1 | 2.174543014694402e-9 | 0.026057 |
| KNN | 0.000026 | 0.000010 | 8.652486120944639e-20 | 2.174543014694402e-9 | 1 | 1.343964344931894e-10 |
| LGBM | 0.000989 | 0.032832 | 0.159385 | 0.026057 | 1.343964344931894e-10 | 1 |

**Supplementary Table S11. Summary of AUC values and their 95% confidence intervals for the training and validation sets**

| model | dfclass | AUC | Low | Up |
| --- | --- | --- | --- | --- |
| LR | dev | 0.881 | 0.861 | 0.902 |
| DT | dev | 0.854 | 0.833 | 0.876 |
| XGB | dev | 0.968 | 0.959 | 0.978 |
| SVM | dev | 0.88 | 0.859 | 0.901 |
| KNN | dev | 0.912 | 0.895 | 0.929 |
| LGBM | dev | 0.94 | 0.926 | 0.955 |
| LR | vad | 0.867 | 0.835 | 0.9 |
| DT | vad | 0.841 | 0.807 | 0.875 |
| XGB | vad | 0.938 | 0.916 | 0.96 |
| SVM | vad | 0.865 | 0.832 | 0.898 |
| LR | dev | 0.881 | 0.861 | 0.902 |
| DT | dev | 0.854 | 0.833 | 0.876 |

**Supplementary Table S12. Brier scores of models in the training set**

| name | bs_score |  |  |  |
| --- | --- | --- | --- | --- |
| xgboost | brier_score= 0.0622 (0.0545-0.0713) | | | |
| SVM | brier_score= 0.1361 (0.1235-0.1484) | | | |
| LR | brier_score= 0.1342 (0.1231-0.1467) | | | |
| lgbm | brier_score= 0.0889 (0.0793-0.1001) | | | |
| KNN | brier_score= 0.1147 (0.1029-0.1285) | | | |
| DT | brier_score= 0.1329 (0.1205-0.1480) | | | |

**Supplementary Table S13. Brier scores of models in the validation set**

| name | bs_score |  |  |  |
| --- | --- | --- | --- | --- |
| xgboost | brier_score= 0.0913 (0.0760-0.1112) | | | |
| SVM | brier_score= 0.1463 (0.1285-0.1660) | | | |
| LR | brier_score= 0.1448 (0.1256-0.1643) | | | |
| lgbm | brier_score= 0.1134 (0.0965-0.1342) | | | |
| KNN | brier_score= 0.2365 (0.2046-0.2681) | | | |
| DT | brier_score= 0.1414 (0.1216-0.1668) | | | |

**Supplementary Table S14. Area under the precision-recall curve (PR-AUC) for each model in the training set.**

| dataset | PRAUC | type |
| --- | --- | --- |
| dev | 0.475 | DT |
| dev | 0.477 | KNN |
| dev | 0.475 | lgbm |
| dev | 0.475 | LR |
| dev | 0.475 | SVM |
| dev | 0.475 | xgboost |

**Supplementary Table S15. Area under the precision-recall curve (PR-AUC) for each model in the validation set.**

| dataset | PRAUC | type |
| --- | --- | --- |
| vad | 0.477 | DT |
| vad | 0.474 | KNN |
| vad | 0.477 | lgbm |
| vad | 0.477 | LR |
| vad | 0.477 | SVM |
| vad | 0.477 | xgboost |

**Supplementary Table S16. Comprehensive performance metrics of the six models in the training cohort.**

| **Model** | **AUC** | **AUPRC** | **Accuracy** | **Sensitivity** | **Specificity** | **PPV** | **NPV** | **F1** | **MCC** | **Kappa** | **Brier** | **LogLoss** | **Youden** |
| --- | --- | --- | --- | --- | --- | --- | --- | --- | --- | --- | --- | --- | --- |
| DT | 0.908 | 0.894 | 0.869 | 0.855 | 0.881 | 0.852 | 0.883 | 0.854 | 0.736 | 0.736 | 0.104 | 0.344 | 0.736 |
| KNN | 0.928 | 0.891 | 0.834 | 0.721 | 0.925 | 0.885 | 0.804 | 0.794 | 0.667 | 0.657 | 0.118 | 0.364 | 0.645 |
| LR | 0.875 | 0.816 | 0.822 | 0.752 | 0.879 | 0.833 | 0.815 | 0.791 | 0.639 | 0.637 | 0.137 | 0.453 | 0.631 |
| LGBM | 0.990 | 0.989 | 0.956 | 0.932 | 0.975 | 0.968 | 0.947 | 0.949 | 0.910 | 0.910 | 0.040 | 0.159 | 0.906 |
| **XGBoost** | **0.968** | **0.960** | **0.918** | **0.871** | **0.956** | **0.941** | **0.902** | **0.905** | **0.835** | **0.833** | **0.065** | **0.236** | **0.827** |
| SVM | 0.958 | 0.961 | 0.907 | 0.887 | 0.923 | 0.903 | 0.910 | 0.895 | 0.811 | 0.811 | 0.072 | 0.257 | 0.810 |

AUC, area under the ROC curve; AUPRC, area under the precision–recall curve; PPV, positive predictive value; NPV, negative predictive value; F1, harmonic mean of precision and recall; MCC, Matthews correlation coefficient; Kappa, Cohen’s κ; Brier, Brier score (lower is better); LogLoss, cross-entropy (lower is better); Youden, Youden’s J index. Threshold-dependent metrics use a 0.5 probability cut-off. The XGBoost row is highlighted.

**Supplementary Table S17. Comprehensive performance metrics of the six models in the independent validation cohort.**

| **Model** | **AUC** | **AUPRC** | **Accuracy** | **Sensitivity** | **Specificity** | **PPV** | **NPV** | **F1** | **MCC** | **Kappa** | **Brier** | **LogLoss** | **Youden** |
| --- | --- | --- | --- | --- | --- | --- | --- | --- | --- | --- | --- | --- | --- |
| DT | 0.863 | 0.799 | 0.831 | 0.827 | 0.835 | 0.802 | 0.857 | 0.814 | 0.660 | 0.660 | 0.136 | 0.556 | 0.662 |
| KNN | 0.886 | 0.838 | 0.805 | 0.659 | 0.923 | 0.873 | 0.770 | 0.751 | 0.612 | 0.596 | 0.143 | 0.599 | 0.582 |
| LR | 0.884 | 0.832 | 0.811 | 0.714 | 0.890 | 0.840 | 0.793 | 0.771 | 0.618 | 0.612 | 0.139 | 0.446 | 0.603 |
| LGBM | 0.934 | 0.906 | 0.876 | 0.845 | 0.901 | 0.873 | 0.878 | 0.859 | 0.749 | 0.748 | 0.093 | 0.318 | 0.746 |
| **XGBoost** | **0.938** | **0.918** | **0.880** | **0.809** | **0.938** | **0.913** | **0.859** | **0.858** | **0.759** | **0.755** | **0.093** | **0.322** | **0.747** |
| SVM | 0.923 | 0.854 | 0.886 | 0.868 | 0.901 | 0.876 | 0.894 | 0.872 | 0.770 | 0.770 | 0.093 | 0.331 | 0.769 |

**Supplementary Table S18. Stratified ten-fold cross-validation performance (mean ± SD) on the full cohort.**

| **Model** | **Cross-validated AUC** | **Cross-validated accuracy** | **Cross-validated F1** |
| --- | --- | --- | --- |
| DT | 0.873 ± 0.024 | 0.846 ± 0.025 | 0.827 ± 0.026 |
| KNN | 0.890 ± 0.015 | 0.821 ± 0.022 | 0.775 ± 0.034 |
| LR | 0.873 ± 0.024 | 0.818 ± 0.025 | 0.784 ± 0.034 |
| LGBM | 0.939 ± 0.014 | 0.889 ± 0.022 | 0.874 ± 0.024 |
| **XGBoost** | **0.934 ± 0.016** | **0.885 ± 0.022** | **0.866 ± 0.027** |
| SVM | 0.925 ± 0.023 | 0.880 ± 0.017 | 0.863 ± 0.021 |

Values are the mean ± standard deviation across ten stratified folds.

**Supplementary Table S19. Computational efficiency of the six models.**

| **Model** | **Training time (ms)ᵃ** | **Inference time (ms)ᵇ** | **Peak memory (MB)** | **Model size (KB)** |
| --- | --- | --- | --- | --- |
| DT | 8.8 | 0.71 | 0.142 | 4.2 |
| KNN | 4.1 | 10.82 | 0.148 | 120.7 |
| LR | 6.7 | 0.61 | 0.153 | 0.9 |
| LGBM | 36.2 | 4.52 | 1.446 | 205.3 |
| **XGBoost** | **29.8** | **1.36** | **0.049** | **65.0** |
| SVM | 87.1 | 8.20 | 0.194 | 45.9 |

ᵃ Wall-clock time to fit one model on the 1,148-patient training cohort, averaged over 5 repetitions. ᵇ Time to score the full 492-patient validation cohort, averaged over 20 repetitions. All models trained in well under 0.1 s and required ≤ 1.5 MB of peak memory, confirming that the entire pipeline is feasible on ordinary clinical hardware without specialized accelerators. The selected XGBoost model was the most memory-efficient gradient-boosting configuration (peak memory < 0.05 MB; serialized size 65 KB), enabling near-instantaneous bedside inference.

**Supplementary Table S20. Diagnostic performance of the prediction model on the original continuous probabilities and binarized results of the training set and validation set**

| datatset | Cutoff | AUC | Accuracy | Sensitivity | Specificity | PLR | NLR | PPV | NPV | Kappa |
| --- | --- | --- | --- | --- | --- | --- | --- | --- | --- | --- |
| Trianset_raw | 0.428 | 0.892 | 0.84 | 0.821 | 0.855 | 5.658 | 0.209 | 0.821 | 0.855 | 0.676 |
| Trianset_binary | 0.428 | 0.838 | 0.84 | 0.821 | 0.855 | 5.658 | 0.209 | 0.821 | 0.855 | 0.676 |
| Validationset_raw | 0.423 | 0.876 | 0.825 | 0.807 | 0.839 | 5.028 | 0.23 | 0.8 | 0.846 | 0.646 |
| Validationset_binary | 0.428 | 0.819 | 0.821 | 0.798 | 0.839 | 4.97 | 0.24 | 0.798 | 0.839 | 0.638 |

**Supplementary Table S21. Logistic regression analysis based on the quartiles of nomoScore**

| name | desc | Non-CSVD (N=634) | CSVD (N=514) | OR (multivariable) | |  |
| --- | --- | --- | --- | --- | --- | --- |
| NomoScore | Q1 | 264 (41.6%) | 23 (4.5%) |  |  |  |
|  | Q2 | 231 (36.4%) | 56 (10.9%) | 2.78 (1.66-4.66, p<.001) | | |
|  | Q3 | 107 (16.9%) | 180 (35%) | 19.31 (11.84-31.48, p<.001) | | |
|  | Q4 | 32 (5%) | 255 (49.6%) | 91.47 (52.10-160.57, p<.001) | | |

**Supplementary Table S22. Diagnostic Assessment of nomoScore**

| datatset | Cutoff | AUC | Accuracy | Sensitivity | Specificity | PLR | NLR | PPV | NPV | Kappa |
| --- | --- | --- | --- | --- | --- | --- | --- | --- | --- | --- |
| Trainset | 295.279 | 0.881 | 0.833 | 0.805 | 0.855 | 5.551 | 0.228 | 0.818 | 0.844 | 0.661 |
| Valiset | 295.279 | 0.813 | 0.815 | 0.794 | 0.832 | 4.727 | 0.248 | 0.79 | 0.835 | 0.625 |

including sensitivity, specificity, accuracy, area under the curve (AUC), and optimal cutoff value.

**Supplementary Table S23. Comparison with representative machine-learning studies for cerebral small vessel disease and related cerebrovascular outcomes.**

| **Study (year)** | **Target / cohort** | **Sample size** | **Best model & approach** | **Best AUC** |
| --- | --- | --- | --- | --- |
| Present study (2026) | CSVD occurrence; hospital cohort | 1,640 | XGBoost + SHAP + nomogram (10 routine variables) | 0.938 (val.) |
| Ref. [11] (2025) | CSVD risk; community | 1,489 | XGBoost/LightGBM, RFE, SHAP, web tool | Highᵃ |
| Ref. [12] (2026) | CSVD diagnosis; EMR | 597 | Stacking ensemble + SHAP (12/23 variables) | 0.881 |
| Ref. [13] (2026) | CSVD-related depression; multicentre MRI | 198 | XGBoost, multimodal MRI + SHAP | 0.958/0.893/0.917 |
| Ref. [14] (2025) | WMH; hospital | — | LASSO logistic nomogram | 0.783/0.762 |
| Ref. [15] (2025) | AIS functional prognosis; WMH radiomics | 202 | Clinical + radiomics nomogram | 0.972/0.939 |
| Ref. [16] (2025) | HICH 6-month recovery | 807 | XGBoost + SHAP | 0.921/0.813 |
| Ref. [17] (2024) | Cerebral oedema after sICH | 215 | GDBT/XGBoost + SHAP/LIME | 0.66 (val.) |

AIS, acute ischaemic stroke; EMR, electronic medical record; HICH, hypertensive intracerebral haemorrhage; sICH, spontaneous intracerebral haemorrhage; WMH, white-matter hyperintensities; val., validation cohort. Where two or three values are shown, they correspond to training/validation(/external) cohorts. ᵃ Reported as high discrimination without a single headline AUC in the source. Relative to prior clinical-variable models, the present model attained the highest validation discrimination using only ten inexpensive, routinely collected predictors, approaching the performance of imaging-dependent pipelines while remaining deployable at the bedside.
